# Scalable inference and identifiability of kinetic parameters for transcriptional bursting from single cell data

**DOI:** 10.1093/bioinformatics/btaf581

**Published:** 2025-10-22

**Authors:** Junhao Gu, Nandor Laszik, Christopher E Miles, Jun Allard, Timothy L Downing, Elizabeth L Read

**Affiliations:** Department of Chemical & Biomolecular Engineering, University of California, Irvine, Irvine, CA, 92617, United States; NSF Simons Center for Multiscale Cell Fate, University of California, Irvine, Irvine, CA, 92697, United States; NSF Simons Center for Multiscale Cell Fate, University of California, Irvine, Irvine, CA, 92697, United States; Department of Biomedical Engineering, University of California, Irvine, Irvine, CA, 92697, United States; NSF Simons Center for Multiscale Cell Fate, University of California, Irvine, Irvine, CA, 92697, United States; Department of Mathematics, University of California, Irvine, Irvine, CA, 92697, United States; NSF Simons Center for Multiscale Cell Fate, University of California, Irvine, Irvine, CA, 92697, United States; Department of Mathematics, University of California, Irvine, Irvine, CA, 92697, United States; Department of Physics, University of California, Irvine, Irvine, CA, 92697, United States; NSF Simons Center for Multiscale Cell Fate, University of California, Irvine, Irvine, CA, 92697, United States; Department of Biomedical Engineering, University of California, Irvine, Irvine, CA, 92697, United States; Department of Chemical & Biomolecular Engineering, University of California, Irvine, Irvine, CA, 92617, United States; NSF Simons Center for Multiscale Cell Fate, University of California, Irvine, Irvine, CA, 92697, United States

## Abstract

**Motivation:**

Stochastic gene expression and cell-to-cell heterogeneity have attracted increased interest in recent years, enabled by advances in single-cell measurement technologies. These studies are also increasingly complemented by quantitative biophysical modeling, often using the framework of stochastic biochemical kinetic models. However, inferring parameters for such models (i.e., the kinetic rates of biochemical reactions) remains a technical and computational challenge, particularly doing so in a manner that can leverage high-throughput single-cell sequencing data.

**Results:**

In this work, we develop a chemical master equation model reference library-based computational pipeline to infer kinetic parameters describing noisy mRNA distributions from single-cell RNA sequencing data, using the commonly applied stochastic telegraph model. The approach fits kinetic parameters via steady-state distributions, as measured across a population of cells in snapshot data. Our pipeline also serves as a tool for comprehensive analysis of parameter identifiability, in both *a priori* (studying model properties in the absence of data) and *a posteriori* (in the context of a particular dataset) use-cases. The pipeline can perform both of these tasks, i.e. inference and identifiability analysis, in an efficient and scalable manner, and also serves to disentangle contributions to uncertainty in inferred parameters from experimental noise versus structural properties of the model. We found that for the telegraph model, the majority of the parameter space is not practically identifiable from single-cell RNA sequencing data, and low experimental capture rates worsen the identifiability. Our methodological framework could be extended to other data types in the fitting of small biochemical network models.

**Availability and implementation:**

All code relevant to this work is available at https://github.com/Read-Lab-UCI/TelegraphLikelihoodInfer, archival DOI: https://doi.org/10.5281/zenodo.16915450.

## 1 Introduction

When experiments on gene expression in cells reached single molecule resolution, it was discovered that the fundamental cellular process of transcription is surprisingly noisy (reviewed in Raj and Van Oudenaarden (2008)). Temporal measurements of individual transcribed mRNA molecules revealed that transcription occurs not smoothly and continuously, but in bursts Golding *et al.* (2005). So-called “intrinsic” biochemical noise, arising from the inherent stochasticity of biochemical reactions occurring in low copy number regimes, can partially account for transcriptional noise Swain et al. (2002). More broadly, important roles for biochemical noise have been identified in numerous cellular processes, including cell fate diversification in development (Dietrich and Hiiragi 2007, Losick and Desplan 2008, Yamanaka *et al.* 2010, Gomes et al. 2011), cellular reprogramming (Desai *et al.* 2021), cancer phenotype switching (Sharma et al. 2010, Gupta et al. 2011), and bacterial antibiotic resistance (Balaban et al. 2004).

Discrete, stochastic chemical reaction models have formed the basis of biophysical theories of gene expression noise (Peccoud and Ycart 1995, Kepler and Elston 2001, Thattai and Van Oudenaarden 2001). The two-state gene expression model (also known as the telegraph model) has been a common baseline framework for understanding gene expression noise. In this model, the promoter of interest stochastically switches between two distinct “on/off” activity states. The switching process could represent transcription factor binding/unbinding (Xu *et al.* 2016), mechanical forces (Tripathi and Chowdhury 2008, Desai *et al.* 2021), large-scale chromatin rearrangement occurring on slower timescales (Miller-Jensen *et al.* 2011), etc. Despite its simplicity, this model has gained traction due to its ability to recapitulate properties of gene expression noise from microbes to mammals, including heavy-tailed transcript distributions with negative binomial shape (Paulsson and Ehrenberg 2000), and the scaling properties of noise with mean gene expression (So *et al.* 2011, Sanchez and Golding 2013).

Inference of kinetic parameters of the telegraph model from experimental data has been undertaken in a number of studies. Many of these involved fitting model-output distributions to experimentally measured mRNA histograms from single molecule fluorescence *in situ* hybridization (smFISH) experiments, either from single-timepoint snapshots (Raj *et al.* 2006, Shahrezaei and Swain 2008, Dey et al. 2015, Gómez-Schiavon *et al.* 2017, Bass *et al.* 2021, Desai *et al.* 2021), or temporal measurements (So *et al.* 2011). Recently, there has also been progress in inferring bursting kinetics from single-cell RNA sequencing data (scRNAseq) (Kim and Marioni 2013, Jiang *et al.* 2017, Larsson et al. 2019, Luo *et al.* 2022, Gorin *et al.* 2023, Tang *et al.* 2023, Ramsköld *et al.* 2024). The underlying concept is that of utilizing fluctuations, as measured across a population at steady-state, to infer dynamics. scRNAseq generates single-cell distributions for the entire transcriptome, in principle enabling comprehensive, genome-wide analysis of bursting kinetics. However, scRNAseq also suffers from significant technical noise (Jiang *et al.* 2022), which makes parameter inference challenging.

Parameter identifiability is a subject that has received increased attention in recent years, including in the area of biochemical reaction modeling (Wieland *et al.* 2021). In general, parameters are considered structurally identifiable when a parameter set maps to a unique model output (Raue *et al.* 2009, Komorowski *et al.* 2011). In the case of unlimited, perfect data, the inference and identifiability problem are dependent on the structure of the physical model itself. ‘Structural identifiability’ is distinguished from ‘practical identifiability’, which can be caused by insufficient data, loss of information, or noise sources affecting measurements, all of which could lead to unacceptably large uncertainty of parameter estimates. In model-guided analysis of scRNAseq, practical identifiability (the focus of this study) is of interest because of technical errors and noise in the measurement technologies, and because the sample size is often limited.

Researchers have leveraged various modeling and inference tools to infer kinetic parameters of the telegraph model from scRNAseq, and many of these have included some type of uncertainty analysis (Kim and Marioni 2013, Larsson *et al*. 2019, Luo *et al.* 2022, Tang *et al.* 2023, Ramsköld *et al.* 2024). However, uncertainty analysis is generally performed in an *a posteriori* manner (i.e. in the context of a particular data set, after the fact) (Wieland *et al.* 2021). In this study, we sought to address the question of whether the parameters of the telegraph model are fundamentally, practically identifiable from snapshot scRNAseq data in general, rather than proceeding from the assumption that they *are* identifiable. To this end, we developed a computationally efficient batched chemical master equation (CME)-based pipeline that performs both *a priori* and *a posteriori* inference and analysis of parameter identifiability, fitting steady-state model distributions to measured population distributions. We found that, in most of the biophysical parameter space for the telegraph model, the parameters are not practically identifiable from snapshot mRNA distributions alone, even with 100% capture rate and large sample size. We assess statistical noise measures—i.e. distribution shape features—that can partly, but not fully, predict the identifiability of kinetic parameters for a given gene. In general, based on the telegraph model, only genes undergoing slow promoter switching have identifiable kinetics. Our pipeline and conceptual framework could be applied to other types of small stochastic biochemical network models and measurement contexts, or adapted to more complex models in the future.

## 2 Materials and methods

### 2.1 Telegraph model and CME solution

**Figure 1.**
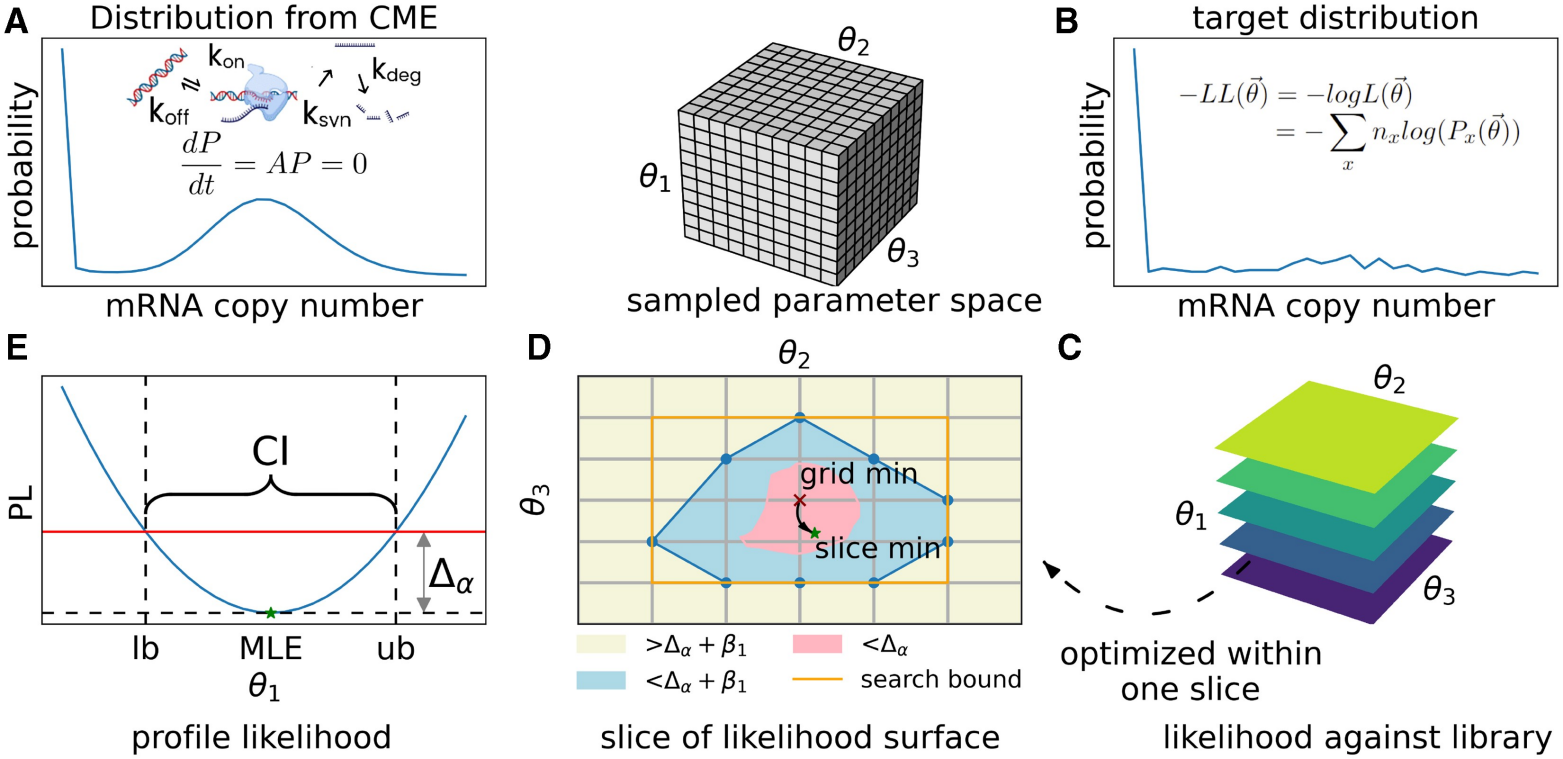
Computational pipeline workflow. (A) mRNA distribution computed from the telegraph model, where the promoter switches between inactive and active states. Parameter sets are sampled as a 3D grid library for the parameters $k_{\text{syn}}$, $k_{\text{off}}$, and $k_{\text{on}}$ (see Section 2). (B) Representative experimentally measured target distribution, from which the negative log-likelihood (−LL) of sampled parameter sets $\vec{\theta }$ can be obtained by comparison to computed distributions. Alternatively, the target distribution can be obtained from synthetic data (i.e. model-generated distributions) for *a priori* identifiability analysis. (C) The coarse-grained, 3D surface, i.e. the −LL value of every simulated mRNA distribution from the model library against the target distribution. (D) A schematic slice from the 3D −LL surface, demonstrating the optimization procedure: optimization is only performed within the search bounds obtained from the initially sampled coarse-grained −LL surface. (E) After optimization, the profile likelihood (PL) function for each parameter is obtained and confidence intervals are computed (see Section 2 and Section SI 1, available as supplementary data at *Bioinformatics* online).

The reactions of the telegraph model are as follows:


$$\begin{array}{c} G\overset{k_{\text{on}}}{\underset{k_{\text{off}}}{⇄}}G^{*}\\ G^{*}\overset{k_{\text{syn}}}{\to }G*+\text{mRNA}\\ \text{mRNA}\overset{k_{d}}{\to }\emptyset \end{array}$$

where $G^{*},G$ represent the states of the promoter of the gene of interest (active, inactive, respectively). The gene state switches between active and inactive with reaction rates $k_{\text{on}}$ and $k_{\text{off}}$, respectively, and mRNA is produced at rate $k_{\text{syn}}$ only in the active state (Fig. 1A). $k_{d}$ is the mRNA degradation rate. We take the inverse degradation rate to be unit time of the system; thus the model is comprised of three parameters to be inferred, expressed by the vector $\vec{\theta }=\{k_{\text{on}},k_{\text{off}},k_{\text{syn}}\}$, all effectively scaled by $k_{d}$, thus setting $k_{d}=1$. Kinetics of the telegraph model are sometimes characterized in terms of transcript burst size $k_{\text{syn}}/k_{\text{off}}$ and burst frequency $\frac{k_{\text{on}}k_{\text{off}}}{k_{\text{on}}+k_{\text{off}}}$.

Although the telegraph model admits an analytical solution (Peccoud and Ycart 1995), we adopt the CME framework because it can also be applied to other models. The system can be expressed in vector–matrix form by enumerating a finite number of states (i.e. neglecting low-probability states) (Munsky and Khammash 2006). The CME is expressed as:


(1) $$\frac{dP}{dt}=AP$$

where $P\equiv P(\vec{s},t)$ is the probability to find the system in system state $\vec{s}$ at time *t*, and *A* is the reaction rate matrix, whose elements $A_{ij}$ give the rate of the reaction bringing the system from state *j* to state *i*, given by the model’s kinetic parameters and standard chemical rate laws. The in-principle infinite state-space is truncated by neglecting low-probability high-mRNA-count states, with errors $\leq 10^{-4}$. The steady-state solution $P(\vec{s},t\to \infty )$ is thus obtained from AP=0 (see Section SI 1.1, available as supplementary data at *Bioinformatics* online, in Supplementary Extended Methods for details on the structure of *A* and truncation method).

For convenience, we hereon refer to the steady-state model solution on the full state-space as $P(\vec{s})$. This solution can be further projected onto the mRNA copy number axis *x*, giving P(x), by summing over the on/off promoter states. This is done because promoter state is not directly distinguishable from scRNAseq data. Scaling by $k_{d}$, there are three inference parameters, as vector $\vec{\theta }=\{k_{\text{on}},k_{\text{off}},k_{\text{syn}}\}$. We hereon refer to the model-computed steady-state distribution over mRNA molecules, given parameter vector $\vec{\theta }$, $P(x|\vec{\theta })$ as $P_{x}(\vec{\theta })$, or $P_{x}^{\text{sim}}(\vec{\theta })$, to further distinguish from an experimentally measured target distribution. Note that, with population distributions from scRNAseq data, and assuming steady-state, it is in principle feasible to infer all three parameters from snapshot data using the stochastic model, depending on the features of the distribution. In contrast, the mean-field ODE model predicts only the steady-state mean, $\langle \text{mRNA}\rangle =\frac{k_{\text{syn}}k_{\text{on}}}{k_{\text{on}}+k_{\text{off}}}$; thus, the parameters are structurally unidentifiable when using only the mean.

### 2.2 Model of measurement noise from dropout

It is experimentally impossible to have 100% of the mRNA captured and amplified in scRNAseq, contributing to the “dropout” problem of excessive zeros in scRNAseq (Jiang et al. 2022). To mimic such a process, our generated mRNA distribution library is modified with a binomial downsampling matrix with different capture rates (Tang *et al.* 2023), which are assumed known. For the telegraph model, this transformation is equivalent to analytically scaling the synthesis rate by the capture efficiency (Tang *et al.* 2020). While this scaling offers computational benefits, our numerical approach is chosen for its broad applicability to more complex models that may lack this property. Hence our modeling incorporates two types of technical error: limited sample size *N* and reduced capture rate (see Section SI 3.2, available as supplementary data at *Bioinformatics* online).

### 2.3 Parameter estimates and confidence intervals

We use a standard approach for *a posteriori* parameter inference, using maximum likelihood estimation (MLE) and profile likelihood (PL) functions to derive confidence intervals (CIs) (Fig. 1E and Section SI 1.2, available as supplementary data at *Bioinformatics* online). For comparison of the model output to mRNA count distributions from scRNAseq, the log-likelihood function over parameter sets $\vec{\theta }$ is:


(2) $$LL(\vec{\theta })=\text{log}\;L(\vec{\theta })=\sum_{x}n_{x}\;\text{log}(P_{x}(\vec{\theta }))$$

where *x* is the mRNA copy number and $n_{x}$ is the number of cells with *x* observed mRNA copies (Fig. 1B) (Note: we use minimization of $-LL(\vec{\theta })$ to obtain MLE).

### 2.4 PL for *a priori* identifiability analysis

We first study the identifiability of the telegraph model in the idealized scenario: when the model itself is used to generate synthetic data. This is an *a priori* approach, because it depends only on the properties of the model itself and not on any particular dataset. For a hypothetical experiment with *N* cells and parameters ${\vec{\theta }}_{\text{tar}}$, Equation (2) becomes


(3) $$H(\vec{\theta })=-N\sum_{x}P_{x}^{\text{tar}}({\vec{\theta }}_{\text{tar}})\;\text{log}(P_{x}^{\text{sim}}(\vec{\theta }))$$

where $P_{x}^{\text{tar}}$ is the model-calculated probability of observing *x* mRNA in the target distribution, and $P_{x}^{\text{sim}}(\vec{\theta })$ is the simulated probability of observing *x* mRNAs, given any parameter set $\vec{\theta }$.

For consistency with the *a posteriori* analysis and standard terminology, we also refer to the surface $-H(\vec{\theta })$ as the “log-likelihood surface”, while noting it is technically a scaled negative cross-entropy. To study identifiability *a priori*, the question is what shape $-H(\vec{\theta })$ has. In the ideal case, it is narrowly peaked, yielding relatively narrow CIs. However, if varying values of $\vec{\theta }$ produce similar *P*, then the surface may have a broad peak with no clear global minimum—thus practically unidentifiable. The utility of Equation (3) is that one can study the effect of the cell number, *N*, in the hypothetical experiment without any error introduced by sampling.

We adapted the PL method to *a priori* analysis, whereas it is typically used in an *a posteriori* manner (Wieland *et al.* 2021), by removing the need to use sampled data via Equation (3) (see Section SI 1.3, available as supplementary data at *Bioinformatics* online). We refer to the PL so obtained as the “ground-truth PL”, equivalent to the average of sample-replicate-derived PL functions from infinite hypothetical experiments with *N* cells.

### 2.5 Computational pipeline

#### 2.5.1 Strategy to combine coarse-grained model library with fine-grained optimization

Obtaining accurate MLE and CI involves non-linear optimization, which can suffer from local minima, early termination, and lack of efficiency, if poor initialization and boundary conditions are given. For a scalable approach (that deals efficiently with large numbers of experimental distributions, e.g. from transcriptome-wide data), we use a coarse-grained, simulated library of CME-derived-distributions as a reference. We hereon refer to this as the “model library”. This provides coarse-grained estimates of MLE, PL, and CI, and also provides reasonable initial guesses and boundary conditions for further optimization (Fig. 1D and Section SI 1.4, available as supplementary data at *Bioinformatics* online).

#### 2.5.2 Generation of the reference model library from the CME

We solved the CME with a grid sampling of the parameter space. For the three parameters, we took a resolution of 60 1D grid points for each parameter: $k_{\text{syn}}:[10^{-0.3},10^{2.3}],k_{\text{off}}:[10^{-3},10^{3}],k_{\text{on}}:[10^{-3},10^{3}]$ (all in units of $k_{d}^{-1}$, ${\;\text{log}\;}_{10}$ spacing) based on typical measured ranges (Schwanhäusser *et al.* 2011, Geertz *et al.* 2012). Using Equation (3), each target parameter set (i.e. the ground truth to be inferred) is thus mapped to a value of the *LL* surface at $60^{3}$ grid points (see Fig. 1C). The same grid points are also used as targets, thus the model library also entails ${\left(60^{3}\right)}^{2}$ precomputed *LL* values.

#### 2.5.3 PL-based identifiability metric

We devised a PL-based metric for identifiability, termed the alternative precision measure (APM):


(4) $$\text{APM}={\;\text{log}\;}_{T}(\theta_{\text{ub}}/\theta_{\text{lb}})$$

where $\theta_{\text{ub}}$ and $\theta_{\text{lb}}$ are the upper bound and lower bound of the CI, respectively, and *T* is user-defined. In general, the smaller the APM, the smaller the CI and the more identifiable the parameter. We apply a simple cutoff: when APM<1, we consider the model (at that point in parameter space) to be practically identifiable. An APM below 1 indicates that the CI ratio falls within the chosen factor *T*. These threshold values (*T*) define a practical criterion for classifying identifiability, based on typical parameter scales and the sensitivity of the model. We used *T* values $\left\{k_{\text{syn}},k_{\text{on}},k_{\text{off}}\}=\{3,100,100\right\}$. We chose a relatively large value of *T* for $k_{\text{on}}$, $k_{\text{off}}$ due to the broadly varying timescales of processes underlying promoter state-switching in mammalian cells [e.g. from binding kinetics (Geertz *et al.* 2012) to chromatin remodeling (reviewed in Raj *et al.* (2006) and Miller-Jensen *et al.* (2011))]. While the precise cutoff is subjective, overall trends in identifiability remain robust across parameter space (Fig. S2, available as supplementary data at *Bioinformatics* online).

To obtain a single APM value at a given point in parameter space, we use the maximum of the three individual parameter APM values, reasoning that the largest value reflects the “worst” parameter, i.e. the one that is least identifiable from the data (note this is similar to a related, recent method that used the union of profile-wise prediction intervals (Simpson and Maclaren 2023)).

We also studied the more common precision metric for comparison, Fig. S1 and Section SI 2.1, available as supplementary data at *Bioinformatics* online.

## 3 Results

### 3.1 Representative cases; PL matches bootstrapping MLEs

**Figure 2.**
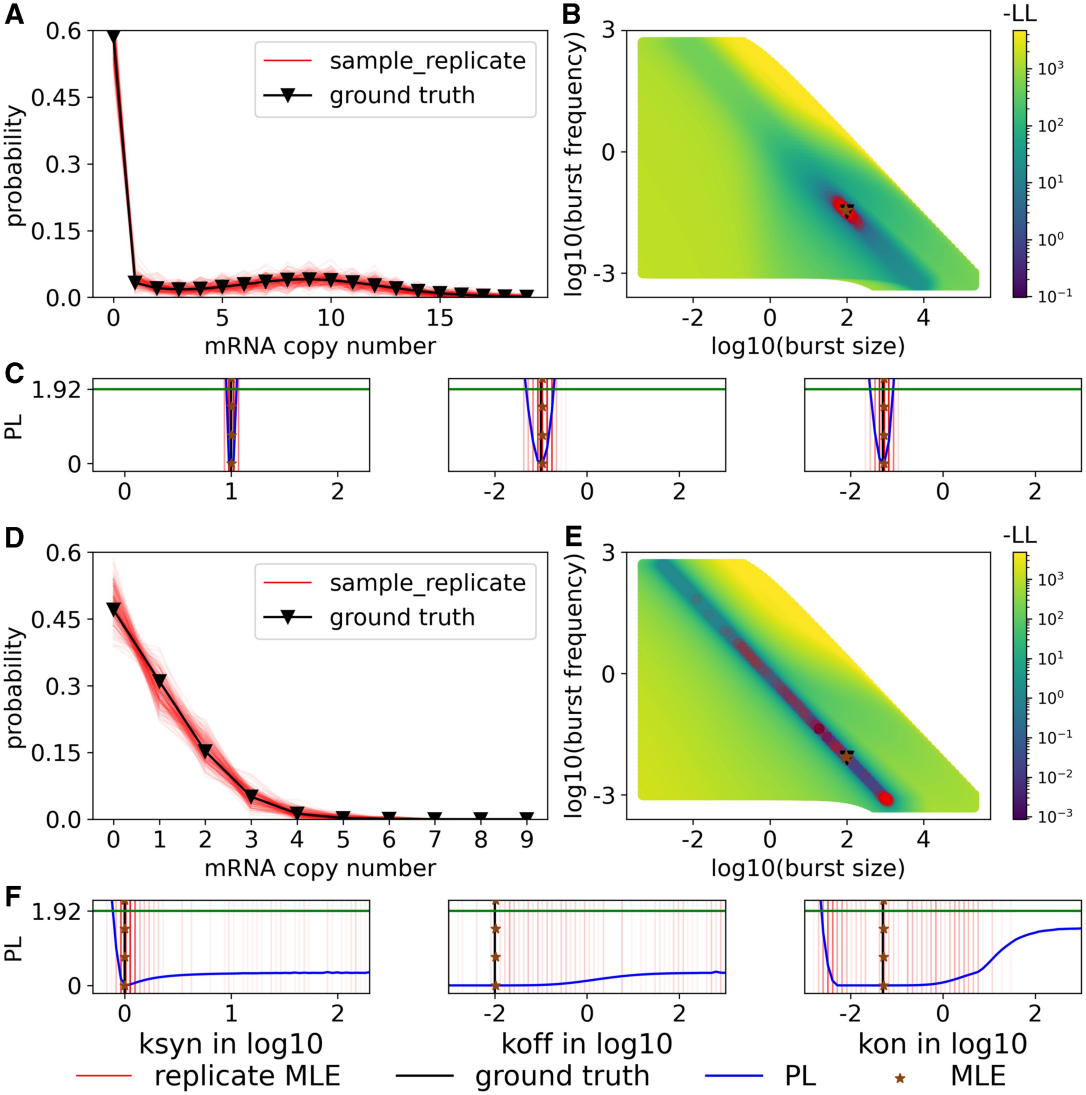
Profile likelihoods (PL) from two representative parameter sets with 200 cells. Panels (A, B, C) Representative parameter set that is identifiable [$k_{\text{syn}}$:10, $k_{\text{off}}$:0.1, $k_{\text{on}}$:0.05], (D, E, F) Representative parameter set that is practically unidentifiable [$k_{\text{syn}}$:10, $k_{\text{off}}$:0.01, $k_{\text{on}}$:0.05]. (A, D) Original computed distributions (black) and sample replicates (red); (B, E) 3D −LL surface projected onto 2D burst frequency and burst size (performed as a scatter plot of sorted −LL values in 2D; when there is overlap, smaller values are in front); (C, F) PL of the three parameters: $k_{\text{syn}},k_{\text{off}},k_{\text{on}}$. For the red dots and stripes, the intensity indicates the frequency of the replicate MLE. The overall PL distribution covers the parameter range where the MLEs take place. The green horizontal lines indicate the 1.92 $\chi^{2}$ value.

We demonstrate the computational approach with two representative parameter scenarios (Fig. 2), one of which is practically identifiable and one which is not. In each case, the ground-truth distribution is shown together with the *LL* surface over the parameter space and the recovered PL in the three separate parameter dimensions. (For ease of visualization, we project the 3D LL surface onto the 2D space of burst frequency and burst size). Additionally, we present results of sampling replicate synthetic distributions, which enables estimation of CIs from bootstrapping.

For the identifiable case (Fig. 2A–C), PL functions are narrow and MLE replicates are narrowly distributed for each parameter. In contrast, in the practically unidentifiable case (Fig. 2D–F), the PL is so broad that either a global minimum is not found, or if found, a majority of the parameter space lies within the confidence region. These scenarios demonstrate how the parameters of the telegraph model may or may not be identifiable, depending on the kinetic regime and sample size.

We observe consistency between the distribution of the sampled MLEs (red) and the ground-truth PL (blue) (Fig. 2C and F), indicating also consistency between the CIs inferred from both methods. The advantage of our *a priori* approach is that it accounts for the loss of identifiability because of finite cells, yet does not contain any error due to sampling (since it represents the limit of finite cells but infinite experiments). Our approach also has the advantage of increasing computational efficiency, since the cell number *N* is simply a scalar multiple of LL and PL. Thus, one does not need to recompute the model library or perform sampling to assess the impact of experimental cell number on identifiability.

### 3.2 Most of the parameter space is unidentifiable

**Figure 3.**
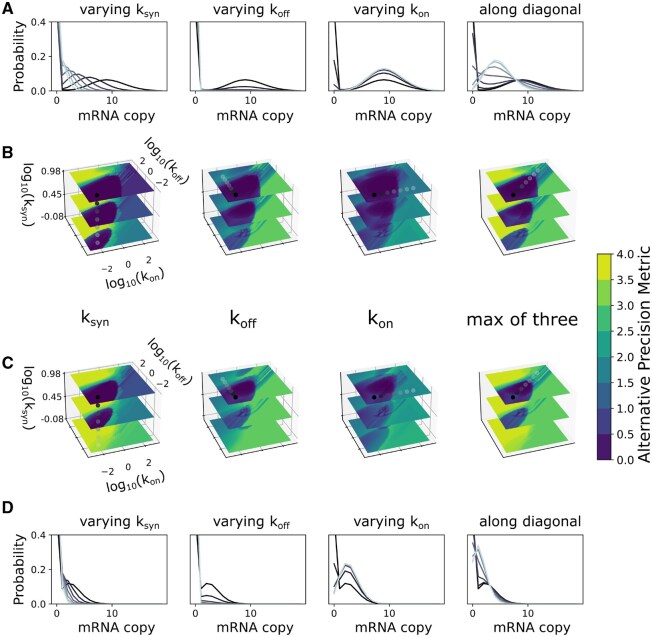
Global *a priori* identifiability landscape over the entire studied parameter space at different capture rates for 10K cells. (A, B) Results for 100% capture rate. (A) mRNA distributions for representative parameter sets. (B) Identifiability (measured by APM) at each ground-truth point in the 3D parameter space of each parameter separately (left three columns) and the overall identifiability (last column, maximum APM from all parameters). Distributions in (A) correspond to dots (grayscale color) in the corresponding 3D surfaces in (B). (C, D) Same as top rows, but with 30% experimental capture rate.

To assess identifiability, we comprehensively computed CIs, and hence APMs, across the parameter space. Figure 3B and C shows the APM (each parameter and combined) for two different experimental RNA capture rates (100%, 30%), over the entire 3D parameter space, for a sample size of $10^{4}$ cells (a realistic *N* value). However, capture rates typically range from 10% to 30%, depending on toolkits used (Bustin *et al.* 2015, Zheng et al. 2017, Schwaber *et al.* 2019, Zucha *et al.* 2020), so these results represent an optimistic scenario. Even so, in Fig. 3, for most of the explored parameter space, the model is not identifiable.

We observed that the region of parameter space showing practical identifiability is shaped like an inverted cone near the slow $k_{\text{on}}$ and $k_{\text{off}}$ regime, indicating that transcriptional bursting kinetics are most identifiable in the slow-gene-switching scenario. The corresponding distributions typically show bimodality at 100% capture rate, as seen in the distributions in darker color in Fig. 3A. Distributions (in lighter color) corresponding to either fast $k_{\text{on}}$ or $k_{\text{off}}$ are more Poisson-like and less identifiable. Note that with smaller $k_{\text{syn}}$, the second mode is at low mRNA count, and only very slow $k_{\text{on}}$ and $k_{\text{off}}$ can practically lead to separation of the two modes and hence identifiability. These results of *a priori* analysis demonstrate that one should be careful with the assumption of identifiability for the telegraph model.

We also studied identifiability as a function of bursting kinetics. Burst-like transcription refers to fluctuations in mRNA synthesis that can be characterized by rapid synthesis (i.e. bursts) followed by periods of relative inactivity, and it has been observed from prokaryotes to mammals (Raser and O’Shea 2004, Golding *et al.* 2005, Raj *et al.* 2006). Transcription burst size (i.e. average number of mRNAs produced in a single burst) and burst frequency are often used to characterize kinetics (Dar *et al.* 2012, Larsson et al. 2019, Bass *et al.* 2021), and these measures may be, arguably, of more interest from a biological standpoint than the kinetic rate parameters themselves. These measures can be directly obtained from parameters in the telegraph model (see Section 2).

However, we noticed that this effective projection of the 3D parameter space $k_{\text{on}},k_{\text{off}},k_{\text{syn}}$ onto 2D (burst size and burst frequency) can be misleading. Systems with the same burst size and burst frequency can differ greatly in identifiability (Fig. S3, available as supplementary data at *Bioinformatics* online). Moreover, much of the (2D) parameter space is again unidentifiable according to APM. Therefore, inference of burst size and burst frequency from scRNAseq via the telegraph model may not be reliable.

### 3.3 Effect of cell number and RNA capture rate

**Figure 4.**
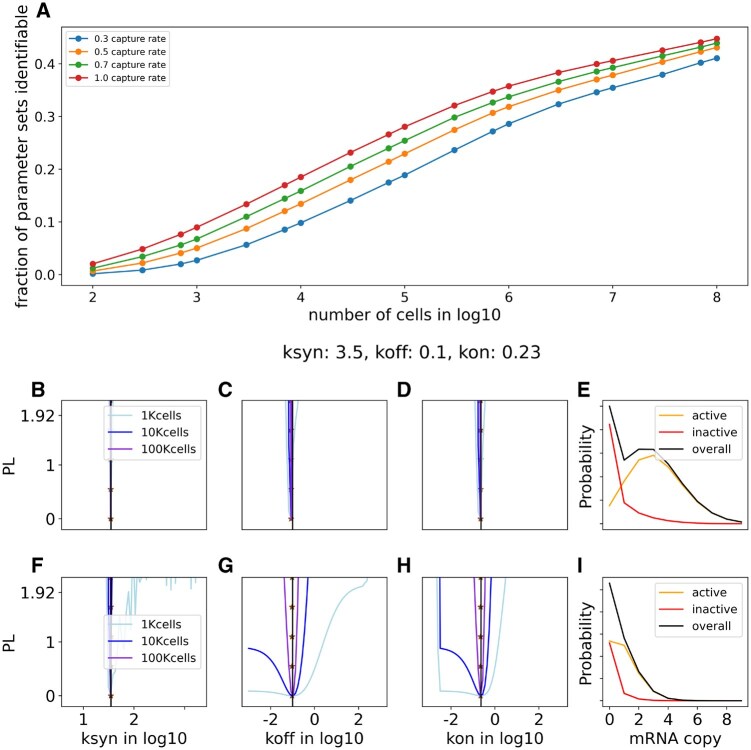
The effect of cell number and capture rate. (A) Fraction of identifiable parameter sets from the whole library grid versus number of cells at different capture rates; (B–D) The profile likelihoods for a representative parameter set $\left\{k_{\text{syn}}:3.5,k_{\text{off}}:0.1,k_{\text{on}}:0.23\right\}$ at 100% capture rate, cell number 1K (light blue), 10K (blue), 100K (violet). (E) The mRNA distribution conditioned on active (G*) and inactive (G) promoter states. (F–I) Corresponding results for the same parameter set as to (B–E) for 30% capture rate.

We explore the effect of capture rate and sample size on identifiability across a range of possible bursting kinetics. We characterize percentage of total parameter sets (from the $60^{3}$-size library grid) that are identifiable for capture rates ranging from 30% to 100% (i.e. at or better than current scRNAseq technologies) and cell numbers ranging from 100 to $10^{8}$. Across this range of cell numbers, to achieve the same percentage of identifiable parameter sets at 30% as compared to 100% capture, would require an increase of 10 times the number of cells, on average. Conversely, for a certain *N*, where $N\leq 10^{4}$, the percentage of identifiable parameter sets is reduced by at least half. The lower *N*, the more drastic the reduction of size of the identifiable parameter space (Fig. 4A). The effect of these technical noise sources on identifiability for specific genes depends on their underlying bursting kinetics. In a scenario with moderately slow switching between promoter states (Fig. 4B–I), approximately 100 times more cells are needed to achieve the same level of precision at 30% capture rate, as compared to 100%.

Bimodality is a key feature of the telegraph model, which is linked to the effects of both *N* and capture rate on identifiability. The bimodality arises from separation between mRNA distribution peaks in the two promoter states, *G*, G*, which occurs when $k_{\text{on}}$ and $k_{\text{off}}$ take low or moderate values. This is seen by computing the mRNA distributions conditioned on each of the two promoter states (Fig. 4E and I). For the scenario in Fig. 4B–E, at 100% capture rate there is clear separation between the modes, resulting in narrow CIs over all parameters, even at the smallest sample size. For the same parameters, mode separation is lost due to signal degradation at lower capture rate (Fig. 4F–I), thus requiring at least 100K cells to achieve identifiability of all parameters. The problem is exacerbated for genes with low transcription rates ($k_{\text{syn}}$), because one can less afford signal degradation when few molecules are present to begin with. Thus, low-$k_{\text{syn}}$ genes require especially slow switching kinetics ($k_{\text{on}},k_{\text{off}}$) for identifiability. Conversely, high transcribing genes have identifiable switching kinetics over a broader range.

Another scenario where low capture rate is especially detrimental is one where gene activity is intermittent, i.e. long periods of inactivity punctured by brief periods of activity. This occurs when both $k_{\text{on}}$ and $k_{\text{off}}$ are slow or moderately slow, and $k_{\text{on}}<k_{\text{off}}$, resulting in bimodality with low probability in the active mode. Here, the model sometimes fails to distinguish between a bimodal distribution (with low probability in the high peak) and a corresponding unimodal distribution.

### 3.4 Partial consistency between PL and other measures for *a priori* identifiability analysis

To validate our findings on the global identifiability landscape based on PL (i.e. in Fig. 3), we compare the results to those obtained from other approaches. One can calculate the sensitivity of the model output to parameter changes directly (i.e. without data), hence sensitivity analysis is an *a priori* approach. The elements of the sensitivity matrix $S_{ji}=\frac{\partial y_{j}}{\partial \theta_{i}}$ give the change of model output $y_{j}$ with respect to parameter $\theta_{i}$. The magnitude of singular values of matrix *S* was suggested as a measure of parameter identifiability in dynamical systems (Sun and Hahn 2006). Other studies utilized similar techniques based on the sensitivity matrix derived from the CME, such as the Fisher Information Matrix (Gunawan et al. 2005, Fox and Munsky 2019).

Our sensitivity analysis, based on the minimum singular value of the sensitivity matrix (Fig. S4A, available as supplementary data at *Bioinformatics* online), supports the telegraph model as structurally identifiable across the explored parameter space, as non-zero singular values were consistently found (albeit very small, $O(10^{-15})$ in some regions). This indicates that the widespread lack of identifiability observed (Fig. 3) is overwhelmingly a *practical* rather than *structural* issue, arising from low model sensitivity combined with finite sample sizes and experimental noise.

The minimum singular value, computed over the studied parameter space, qualitatively resembles the overall output of our PL-pipeline output (Fig. S4A, available as supplementary data at *Bioinformatics* online). Specifically, the region of increased sensitivity occupies the slow-promoter-switching kinetic region (low $k_{\text{off}}$ and $k_{\text{off}}$), and the size of this region increases with $k_{\text{syn}}$. These results demonstrate that the PL is a viable way to access similar information to the sensitivity. However, our PL-based approach has the advantage that the effect of cell number and capture rate can be included in the analysis, rendering it a more pragmatic approach.

We further explored how various RNA-distribution summary statistics varied as a function of the parameter values, reasoning that it could be useful if simple summary statistics correlated with identifiability, thus providing an alternative means of *a priori* analysis. We studied various summary statistics based on moments of the distribution, including the Fano factor $\sigma^{2}/\mu$ (often used as a measure of dispersion in gene expression) (see Figs S4 and S5, available as supplementary data at *Bioinformatics* online). In general, measures related to second or third moments correlated, albeit only somewhat, with identifiability. For example, the Fano factor can be relatively insensitive to bimodality when one mode has very low probability (further details in Section SI 2.2, available as supplementary data at *Bioinformatics* online). Thus, no single shape metric studied could replace the PL-based pipeline as a predictor of identifiability.

### 3.5 *A posteriori* parameter inference from data: few genes have identifiable bursting kinetics

**Figure 5.**
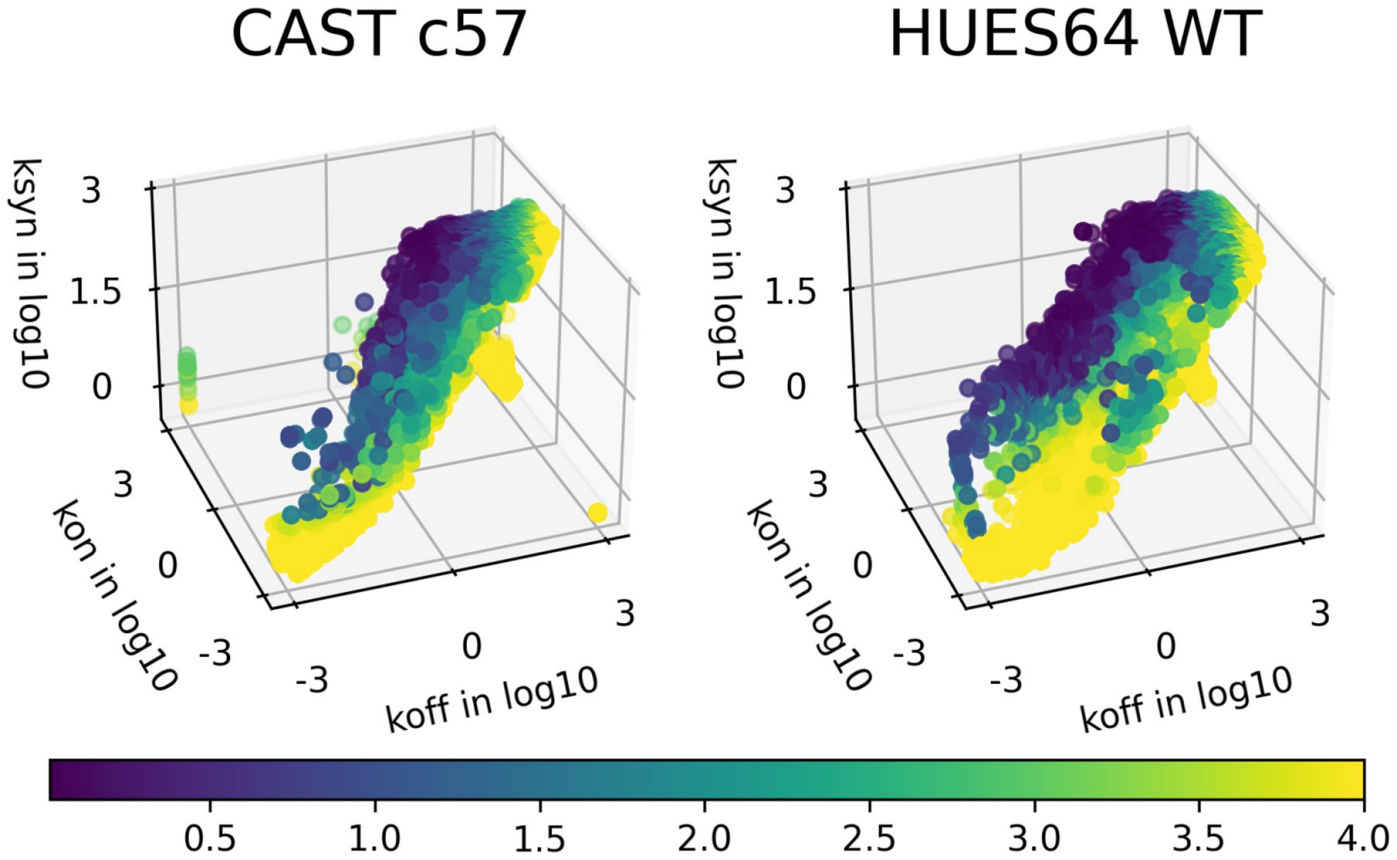
Inferred kinetic parameters of the telegraph model based on two datasets: SS3 cast of mouse fibroblast (CAST/EiJ × C57BL/6J) with cell numbers ranging from 6 to 224 (with mean 208), and HUES64 human embryonic stem cell with cell number of 1112. Each dot corresponds to a gene in the dataset. Color indicates the identifiability of the gene, as quantified by the APM metric derived from profile likelihood-based CIs (maximum over all three parameters). Only a small fraction of genes can be inferred with narrow CI, and thus reach the criterion of identifiability (APM<1).

We applied the PL-based inference pipeline to two scRNAseq datasets: SS3 cast of mouse fibroblast (CAST/EiJ × C57BL/6J) (Larsson et al. 2019) and HUES64 human embryonic stem cells (Charlton et al. 2020). We found that only a small fraction of individual genes’ kinetic parameters are identifiable, according to our criterion (maximum APM from all parameters <1). Nine hundred and seventy gene distributions out of 10 700 genes are considered to have identifiable kinetics for SS3 data, while 1153 genes out of 18 806 are identifiable for HUES64WT (Fig. 5). As expected from the *a priori* analysis, genes for which parameters were inferred with high confidence tend to lie in regions of the parameter space with high expression rates and slow to moderate promoter-switching kinetics. In summary, these results demonstrate that identifiability of transcriptional bursting kinetics from snapshot scRNAseq data is generally poor. Thus, these *a posteriori* results are in general agreement with the findings from *a priori* analysis.

Since identifiability is related to distribution shape, we asked whether it would be possible to rapidly assess identifiability for a given experiment-derived mRNA distribution (together with information on cell number and capture rate) without applying the full PL-based inference pipeline. A neural network was trained on synthetic data to predict APM based on various distribution summary statistics (Section SI 2.3 and Fig. S6, available as supplementary data at *Bioinformatics* online) We then applied the neural network predictor to the real-world datasets, in order to assess its performance.

In general, there was good overlap between the set of identifiable genes (via PL) and those via the neural network. However, many genes were falsely predicted by the neural network to have identifiable kinetics. (Details and discussion of the false-prediction scenarios in Section SI 2.3, available as supplementary data at *Bioinformatics* online). These results demonstrate that, for the real-world data as well as the synthetic data, combinations of distribution summary statistics (as leveraged by the neural network) can only partially predict the identifiability of the telegraph model parameters.

## 4 Discussion

We developed a pipeline to infer kinetic parameters describing noisy gene expression from transcript distributions, such as those obtained from snapshot scRNAseq. Our approach differs from recent, related studies and methods (Kim and Marioni 2013, Jiang *et al.* 2017, Larsson et al. 2019, Luo *et al.* 2022, Tang *et al.* 2023, Ramsköld *et al.* 2024) in two key respects. First, we comprehensively analyse the practical identifiability of kinetic parameters of the classic telegraph model from single-cell data, whereas many related studies report inferred kinetics without critically assessing the identifiability (or lack thereof) of parameters. Second, our pipeline is efficient and scalable: it integrates a batched CME solver with the PL method to quantify parameter uncertainty, utilizing a reference model library of solutions to the CME model. This reduces reliance on optimization and redundant calculations, making the pipeline amenable to analysis of high-throughput transcriptomic data. Another unique aspect of our approach is that we apply the PL method both to *a priori* investigation of identifiability and *a posteriori* inference of parameters; this ensures that the *a priori* study is more directly applicable to the practical output that researchers seek in conducting parameter inference. To this end, our pipeline also incorporates experimental parameters (cell number, capture rate) into the *a priori* analysis. We find that it is often more beneficial to increase capture rate, rather than cell number, to increase identifiability of parameters.

Our results show that the major part of the biologically feasible parameter space for the telegraph model is not practically identifiable from mRNA distributions, according to a criterion that we developed based on precision of inference. We confirmed the result using representative scRNAseq datasets. Sensitivity analysis showed that the model is structurally identifiable, even while practical identifiability is low (echoing previous results on the structural identifiability of a related telegraph model with protein translation (Cinquemani 2018)). In all, these findings underscore the potential pitfalls associated with attempting to infer complex stochastic dynamics from datasets with few degrees of freedom (as in, one dimensional distributions). Despite these potential pitfalls, our study also highlights how even 1D mRNA distributions can, in certain cases, have subtle but distinctive features that can be leveraged by the CME-based inference pipeline to elucidate underlying kinetics, especially in the slow-promoter-switching regime, which is associated with bistable transcript distributions. Identifiability of parameters in this regime has been established previously (Kim and Marioni 2013, Gorin *et al.* 2023). We also find that “borderline” (not clearly bistable) cases can be identifiable (e.g. for moderate promoter switching, low transcription rate, and/or high dropout due to low capture rates in experiments). Such cases can introduce subtle but detectable shifts from Poisson, which can be utilized by the pipeline. Simple summary statistics, such as the commonly used Fano factor, often fail to recognize these borderline cases; as such, this argues for the telegraph model parameters as a more detailed list of shape features to describe noisy gene expression.

Our use of a CME model library, for comparison of the model output to target distributions, is possible because the telegraph model is “small”, i.e. it is a biochemical network model with a limited feasible state-space and a small number of parameters, rendering both the size of the enumerated transition rate matrix, and the size of the feasible parameter space, to be tractable. A number of recent, related studies have combined Bayesian methods with stochastic simulation (Lillacci and Khammash 2013, Zechner *et al.* 2014, Lenive *et al.* 2016, Koblents *et al.* 2019, Molyneux and Abate 2020, Li *et al.* 2021, Fu *et al.* 2022, Jørgensen *et al.* 2022, Trigo Trindade and Zygalakis 2024). The advantage of our approach lies in the relative efficiency of CME solution for small models. Furthermore, the generated model library can be used as reference to decide whether optimization is needed, in contrast to Bayesian approaches, where output distributions are calculated once the parameter prior is sampled, and discarded after the posterior is obtained. However, in use-cases with a small number of target distributions (i.e. few genes), and a more complex model (high-dimensional parameter space), using Bayesian-based methods may be more logical. Nevertheless, our approach could be scaled to more complex biochemical models: as the dimension of model parameters increases, there comes a combinatorial increase of parameter sets. Keeping a manageable sized model library would require a coarser parameter grid and thus more iterations to obtain CI.

The telegraph model has been widely used to describe gene expression noise, although many studies have also noted its shortcomings. It has been successful, e.g. in describing statistical properties of transcript distributions (Paulsson and Ehrenberg 2000, So *et al.* 2011, Sanchez and Golding 2013) and elucidating the link between genomic features and gene expression noise (Larsson et al. 2019). Limitations of the model include its inability to describe biological mechanisms such as downstream processes (Xu *et al.* 2016, Ham *et al.* 2021), feedback regulation (Jiao *et al.* 2024), polymerase dynamics (Cao *et al.* 2020), multiple (>2) promoter states (Munsky *et al.* 2012), and more. The applicability (or not) of the telegraph model to describe real sources of noise in gene expression was not a focus of the present work. Instead, we focused on whether the parameters are identifiable, even in the idealized case, where the model perfectly describes the process to be inferred. Our results support a cautious application of the model to scRNAseq data: the parameters are often not identifiable, but may be for select cases. Our results suggest that more complex models will likely fail to be identifiable from the same type of data, since the issue of inferring multiple parameters from few-degree-of-freedom data would be even more pronounced. This also means that parameter identifiability should be carefully accounted for when comparing accuracy of different models to describe snapshot single-cell data. Despite its shortcomings, the simplicity of the telegraph model may render it more appropriate for inferring noise properties from snapshot 1D distributions than other models, whereas more complex (and biologically descriptive) models should be applied when additional data types are available.

Furthermore, establishing practical thresholds for parameter identifiability, such as our APM criterion, is crucial for downstream biological interpretation. Quantifying how transcriptional kinetics change in response to perturbations (Senecal *et al.* 2014), or defining cell types based on underlying biophysical dynamics (Chari *et al.* 2024), requires parameter estimates with sufficient precision. Our analysis reveals the fundamental resolution limits imposed by the telegraph model when applied to snapshot mRNA distributions alone, suggesting that these data often lack the power to reliably distinguish subtle kinetic changes or classify states based solely on these parameters (i.e. APM is often >1). Achieving the necessary parameter resolution for robust biological interpretation likely requires incorporating richer data types or spatial context that provide additional constraints. This could involve leveraging time-series measurements (Golding *et al.* 2005, So *et al.* 2011, Jiao *et al.* 2024, Nicoll et al. 2025), integrating multimodal single-cell data such as nascent and mature transcript counts (La Manno et al. 2018, Gorin *et al.* 2023, Chari *et al.* 2024), or accounting for the broader biological importance and specific modeling of mRNA spatial dynamics (Engel et al. 2020, Miles 2025). These approaches move beyond the basic assumptions or data limitations explored here, highlighting the need for layered or spatially-resolved data to robustly connect bursting kinetics to cellular function.

## Supplementary Material

btaf581_Supplementary_Data
