# Supplementary material for: Scalable inference and identifiability of kinetic parameters for transcriptional bursting from single cell data
Source: Bioinformatics. 2025 Oct 22;41(11):btaf581. doi: 10.1093/bioinformatics/btaf581 (PMC12646643; doi:10.1093/bioinformatics/btaf581)
Supplement: btaf581_Supplementary_Data [file btaf581_supplementary_data.pdf]

Supplementary Information to:

Scalable Inference and Identifiability of Kinetic Parameters for Transcriptional Bursting from Single Cell Data

SI 1. Extended Methods

SI 1.1. Chemical Master Equation solution

We enumerate the Chemical Master Equation (CME) for the telegraph model as follows. Assuming that the number of mRNA molecules x is always $0 \leq x \leq x_{\max}$, then the number of possible states of the system is $M = 2 \times (x_{\max} + 1)$. (Corresponding to the instantaneous promoter state (on or off) and the number of mRNAs). In principle, the number of mRNA copies in the cell can be any non-negative integer. Practically, it is unlikely for the mRNA to ever greatly exceed k_{syn} (i.e., the synthesis rate scaled by the degradation rate). We assume a value of $x_{\max} = \max\{4\sqrt{k_{\text{syn}}} + k_{\text{syn}}, 25\}$. This $x_{\max}$ definition ensures low error (as the total probability of the truncated states is small) and is convenient for batch calculations. (More precisely, a gene at maximal activity ($k_{\text{on}} \gg k_{\text{off}}$) has Poisson-distributed expression with mean k_{syn}, and the truncated probability under this definition is $<10^{-4}$. For genes with lower activity, the truncated probability is lower.) Note this truncation method is conceptually related to the Finite State Projection Method [3].

The CME is expressed as

$$\frac{dP}{dt} = AP \tag{1}$$

where $P \equiv P(\vec{s}, t)$ is the probability to find the system in system state $\vec{s}$ at time t, and A is the reaction rate matrix, whose elements A_{ij} give the rate of the reaction bringing the system from state j to state i, given by the model's kinetic parameters and standard chemical rate laws. The steady state solution $P(\vec{s}, t \to \infty)$ is thus obtained from $AP = 0$.

The reactions of the telegraph model are as follows:

$$\mathrm{G} \underset{\mathrm{k_{off}}}{\overset{\mathrm{k_{on}}}{\rightleftharpoons}} \mathrm{G}^*$$

$$(\mathrm{G} \xrightarrow{\mathrm{k_0}} \mathrm{G} + \mathrm{mRNA})$$

$$\mathrm{G}^* \xrightarrow{\mathrm{k_{syn}}} \mathrm{G}^* + \mathrm{mRNA}$$

$$\mathrm{mRNA} \xrightarrow{\mathrm{k}_d} \varnothing$$

(Note that here we detail a slightly more complicated version of the telegraph model than that described in the Main Text, allowing for transcription in the "inactive", G promoter state at some non-zero rate k_0. However, in this study we assume the basic form of the telegraph model, i.e., $k_0 = 0$).

Let P_k be a vector of probabilities for the system to be found with mRNA copy number x_0,...,x_n and the promoter to be found in gene state k. The mRNA boundaries of the system correspond to $x_0 = 0$ and $x_n = x_{\max}$, chosen in the manner described above. The system can be written in block matrix form:

$$\frac{d}{dt}\begin{bmatrix} P_{G^*} \\ P_G \\ error \end{bmatrix} = \begin{bmatrix} A_1 & k_{\text{on}} I & 0 \\ k_{\text{off}} I & A_0 & 0 \\ B_1 & B_0 & 1 \end{bmatrix} \begin{bmatrix} P_{G^*} \\ P_G \\ error \end{bmatrix} \tag{2}$$

where I is the identity matrix. The error term absorbs all the degradation and production probability from the two boundary at x_0 and x_n respectively. The matrix A_k is a tridiagonal matrix denoting the connections within states in P_k:

$$A_k = \begin{bmatrix} -L - k_k - k_d x_0 & k_d x_1 & 0 & \cdots & 0 & 0 & 0 \\ k_k & -L - k_k - k_d x_1 & k_d x_2 & \cdots & 0 & 0 & 0 \\ \vdots & \vdots & \vdots & \ldots & \vdots & \vdots & \vdots \\ 0 & 0 & 0 & \cdots & k_k & -L - k_k - k_d x_{n-1} & k_d x_n \\ 0 & 0 & 0 & \cdots & 0 & k_k & -L - k_k - k_d x_n \end{bmatrix}$$

$$B_k = \begin{bmatrix} k_d x_0 & 0 & \cdots & k_k \end{bmatrix}$$

where A_1 tracks state-transitions while the system is in promoter state G^* and A_0 tracks state transitions in G. k_k is the transcription rate of gene state k (i.e., k_{syn} in A_1 and $k_0 = 0$ in A_0). L captures promoter state-switching; $L = k_{\text{off}}$ if A_k is A_1, or k_{on} if A_k is A_0. Note that here we have detailed the structure of the CME including the mRNA degradation rate, k_{d}. Whereas, in the main text we scaled all other rate parameters by k_{d}, effectively setting $k_d = 1$ in the above matrices.

Block representation is used to reduce the matrix size by half. For details on the reduction method, please refer to SI 3.1.

SI 1.2. Parameter estimates and confidence intervals

Maximum Likelihood Estimation

For comparison of the model output to mRNA count distributions from scRNAseq, the log-likelihood function over parameter sets $\vec{\theta}$ is:

$$LL(\vec{\theta}) = \log L(\vec{\theta}) = \sum_x n_x \log(P_x(\vec{\theta})) \tag{3}$$

where x is the mRNA copy number and n_x is the number of cells with x observed mRNA copies (Fig. 1B). The maximum likelihood estimation (MLE) $\widehat{\theta}$ is then the infimum of Eqn. 3. We adhere to the use of minimization:

$$\widehat{\theta} = \underset{\vec{\theta} \in \Theta}{\text{argmin}} \, [-LL(\vec{\theta})] \tag{4}$$

Profile Likelihood-based Confidence Intervals

Profile Likelihood (PL) is often used when accurate confidence interval estimates are difficult to obtain using standard methods, for instance, when LL is non-normal or when the model has high parameter dimensions[4]. PL is also useful for estimating confidence intervals (CI) for each parameter separately. Suppose D is sample data, and the fitted probability mass function is $f(\vec{\theta})$. The parameter space can be partitioned as $\vec{\theta} = (\theta_0, \theta_1)$, where θ_0 is the parameter(s) of interest. The negative log-likelihood function (-LL) can be written as:

$$-LL(\vec{\theta}) = \sum_x -D_x \log f_x(\theta_0, \theta_1) \tag{5}$$

To get the profile likelihood of θ_0, Eqn. 6 is evaluated :

$$\hat{\theta}_1 | \theta_0^i = \underset{\theta_1}{\text{argmin}} \; - LL(\theta_1 | \theta_0^i) \tag{6}$$

This is essentially optimizing θ_1 for the minimum of $-LL$ at different θ_0 values, see Fig. 1D. The minimum of the PL is then subtracted, as the PL evaluates the likelihood ratio of different parameter sets with respect to the minimum $-LL$. The resulting PL $PL(\theta_0)$ is a curve with minimum value at 0:

$$PL(\theta_0) = -\log \frac{L(\theta_1^i | \theta_0)}{L^{min}(\theta)} = -LL(\theta_1^i | \theta_0) + LL^{min}(\theta) \tag{7}$$

By Wilks' theorem, the distribution of likelihood ratio asymptotically approaches χ^2 distribution with increasing total number of cells [4, 6]. Therefore, the χ^2 value (two tail: 1.92, half of 3.84 at one tail) at 1 degree of freedom at some significance level (α=0.05) was used to find the upper bound (θ_{ub}) and lower bound (θ_{lb}) for the CI on parameter θ_0 from $PL(\theta_0)$, as shown in Fig. 1E.

SI 1.3. Profile Likelihood for **a priori** identifiability analysis

We first study the identifiability of the telegraph model in the idealized, best case scenario: when the model itself is used to generate synthetic data. (We also call this 'self-inference'). This is an *a priori* approach, because it depends only on the properties of the model itself and not on any particular dataset.

One can proceed directly from Eqn. 3, where n_x (the number of cells having x mRNA copies) is obtained from synthetic data, i.e., sampled from the CME-model-generated distribution (or, as is often done, simulated by SSA[2]). However, the likelihood then suffers from sampling error. This can be circumvented as follows: Let $\vec{\theta}_{tar}$ denote a target set of parameters, i.e., the set of parameters used to compute the 'target' distribution, which generates the synthetic data, and which we wish to infer. Then $\langle n_x \rangle$, the expectation of n_x, is $NP_x^{tar}(\vec{\theta}_{tar})$, with N being the number of cells in this hypothetical experiment, and $P_x^{tar}(\vec{\theta}_{tar})$ being the target distribution computed from the CME, as above.

In this way, Eqn. 3 can be further factored out the total cell number, N, giving

$$H(\vec{\theta}) = -N \sum_x P_x^{tar}(\vec{\theta}_{tar}) \log(P_x^{sim}(\vec{\theta})) \tag{8}$$

where P_x^{tar} is the model-calculated probability of observing x mRNA in the target distribution, and $P_x^{sim}(\vec{\theta})$ is the simulated probability of observing x mRNAs, given any parameter set $\vec{\theta}$. Eqn. 8 defines the cross-entropy between the target distribution P_x^{tar} and the simulated distribution P_x^{sim}, scaled by the number of cells N. It is minimized at $\vec{\theta} = \vec{\theta}_{tar}$, where it equals the scaled entropy of the target. The curvature near this minimum determines parameter identifiability: it corresponds to the Fisher Information Matrix (FIM), which bounds the variance of unbiased estimators. For consistency with the *a posteriori* analysis and standard likelihood terminology, we refer to the surface $-H(\vec{\theta})$ as the "log-likelihood surface," while noting it is technically a scaled negative cross-entropy.

To study identifiability *a priori*, the question is what shape the hypersurface (Eqn. 8) has. In the ideal case, it is narrowly peaked, yielding relatively narrow CIs. However, if varying values of $\vec{\theta}$ produce similar P, then the surface may have a broad peak with no clear global minimum–thus practically unidentifiable. The utility of Eqn. 8 is that one can study the effect of the cell number, N, in the hypothetical experiment on the LL, without any error introduced by sampling. CIs for each parameter can then be obtained from the LL surface using PL, as above.

We combine this *a priori* estimation of PLs (and hence CIs) with a computationally efficient method of globally scanning the parameter space, as a means to holistically study the identifiability of the parameters of the telegraph model.

Just as the LL hypersurface can be computed directly from P^{tar} for an assumed cell number N without sampling (Eqn. 8), the PL can in turn be obtained in each parameter dimension without sampling. The blue curves in Fig. 2C,F are the PL obtained from ground-truth distributions, given an experiment with N cells. We refer to this as the 'ground truth PL'. Each sample replicate (in red) can be considered to represent one experiment with finite (N) cells, whereas the ground truth PL is equivalent to the average of the sampled distributions' PL, assuming an infinite number of experimental replicates with N cells, as illustrated here:

$$PL(\theta_0|D^j) = \sum_x n_x^j \log(P_x^j(\theta_0|\theta_1^*)) \tag{9}$$

$$PL(\theta_0|D) = \frac{1}{N_j}\sum_j PL(\theta_0|D^j) \tag{10}$$

where j is the replicate/experiment index, N_j is the total number of experiments, and D_x^j is the data sampled from the ground truth distribution in experiment j.

SI 1.4. Computational pipeline

Generation of the reference library from the CME

We use a simulated library of distributions as a reference to compute PLs for assessment of CIs/identifiability. To this end, To accelerate the computation, parameter sets were categorized by the maximum mRNA value based on k_{syn} so that the corresponding transition matrices generation and eigenvalue decomposition were performed batch-wise in numerical arrays, greatly reducing time for library generation. (For further details on how batch-wise/tensor format operations accelerate the library generation process, refer to SI 3.1). For the library used in this work, roughly 20 minutes were used in batch-wise/tensor operation, whereas it took roughly 2 hours in parallel in loop with an 8 core 16 threads laptop.

Strategy to combine coarse-grained library with fine-grained optimization

Obtaining accurate estimates of MLE and CIs involves non-linear optimization, which can suffer from local minima (termination point depends on initial guess), and the early termination problem (if the change in objective function is small per iteration because of a small gradient). It may also require many iterations to find the optimum if poor initial guesses and boundary conditions are given. This may not be a great concern if one is optimizing for one gene distribution, but it is not scalable when dealing with a large number of distributions, e.g. from transcriptome-wide data. To circumvent the poor initial value and boundary condition problems, our use of a coarse-grained, simulated library of distributions as a reference provides reasonable initial guesses and boundary conditions for optimization. Furthermore, the preliminary PL computed against the reference library provides a coarse-grained approximation. A larger/finer-resolution library provides more accurate coarse-grained PL curves and derived CI estimates, thus requiring less computation for latter optimization, at the cost of larger memory usage and time taken to generate the library.

The library is also used to rapidly assess whether further optimization is needed. Regions of the parameter space that are relatively insensitive to parameter changes have flat PLs, and thus wide CIs, and typically take more iterations for optimization. The preliminary PL, computed from these coarse-grained grid points, gives a rough idea of how identifiable each parameter is. Only distributions that are considered potentially identifiable would proceed to further optimization, under the rationale that precise quantification of very large CIs is generally not warranted, as long as these cases can be correctly labeled as 'unidentifiable'. The criterion for further optimization is:

$$\frac{\theta_{0,ub}^i|\Delta_\alpha}{\theta_{0,lb}^i|\Delta_\alpha} < T + \beta_0. \tag{11}$$

where $\theta_{0,ub}^i$ is the initial estimate of the upper bound on parameter θ_0^i, as determined by the coarse-grained PL (similar for $\theta_{0,lb}^i$). Δ_α is χ^2 critical value, 1.92 at 1 degree of freedom, 5% significance level(α), two-tail. T is a threshold that depends on the order of magnitude of the feasible parameter range, and denotes an "acceptable" width of the CI on that parameter (see also next section). β_0 is a buffer parameter that effectively makes the optimization criterion more generous. For example, it can serve to compensate for coarseness of the library grid, especially when the likelihood surface may have a steep gradient rendering initial CI estimates less accurate. As such, it is a user-defined parameter that can depend on factors such as library coarseness and sample size.

Following the same logic, if the optimization criterion is met, then the preliminary 2D likelihood plane also provides the boundary values and the initial guesses of the other parameter inputs for optimization. Optimization is performed within parameter bounds determined by the coarse-grained library as:

$$PL(\theta_1^*|\theta_0^i) < \Delta_\alpha + \beta_1. \tag{12}$$

That is, optimization is performed within the region of parameter space where the initial library-computed PL is within Δ_α plus β_1 of the minimum $-LL$. The value of β_1 depends on cell size, as the log likelihood value is amplified by cell size, for larger cell number, a larger β_1 should be used (-LL is far larger than Δ_α). This potentially circumvents early termination at local minima if any, requires fewer iterations, and improves smoothness of the PL function in the vicinity of the MLE, promoting more accurate estimate of CIs. A non-linear optimization scheme, L-BFGS, was used to find the optimum. Though the $-LL$ function does not have a specific form of gradient with respect to model parameters, using finite difference to approximate the gradient still allows quick convergence.

Profile Likelihood-based identifiability metric

In order to analyze the identifiability of the model itself, i.e., not specific to any assumed local parameter values (as in Fig. 2), but more globally over a broad region of feasible parameters, we first devised a PL-based metric for identifiability. A common measure is the Precision, defined as:

$$\text{Precision} = \log_{10} \frac{\theta_{ub} - \theta_{lb}}{\hat{\theta}} \quad (13)$$

In general, the smaller the Precision, the smaller the CI and the more identifiable the parameter. We also defined another metric, termed the Alternative Precision Measure (APM):

$$\text{APM} = \log_T(\theta_{ub}/\theta_{lb}) \quad (14)$$

where T is a threshold value that is chosen depending on the assumed size of θ's feasible parameter space. We used values of T for $\{k_{\text{syn}}, k_{\text{on}}, k_{\text{off}}\} = \{3, 100, 100\}$ (units of inverse degradation rate). The rationale for the different values is as follows: k_{syn} has a more linear effect on peak location, so the ratio between upper and lower CI bounds tends to be $\mathcal{O}(1)$. In contrast, effects of variation in k_{off} and k_{on} tend to be more non-linear, and the parameters span several orders of magnitude. T sets the value of the ratio of the upper bound to lower bound at which $APM = 1$, i.e., an APM value of 1 for k_{off} indicates that $\theta_{ub} = 100\theta_{lb}$.

Note that the APM is not scaled by $\hat{\theta}$ (unlike Precision). This ensures that a bad estimate of $\hat{\theta}$, in cases of low practical identifiability, does not skew the result. In our testing, the APM provides a better measure of error as compared to Precision, since we are interested in quantifying large errors in order to fairly compare the practical identifiability of the model across different regions of parameter space. For further examples and discussion, see SI 2.1. APM will be the default identifiability metric of use, though we present results for both metrics.

SI 1.5. Variables and Metaparameters

LL	log likelihood
$\vec{\theta}$	parameter vector
$\hat{\theta}$	maximum likelihood estimation of parameter vector
D_x	sample cells with x mRNA transcript
$f(\vec{\theta})$	probability mass function generated by parameter set $\vec{\theta}$
PL	profile likelihood, projection/maximum likelihood along certain dimension(s)
$\Delta\alpha$	statistical significance level at 1 degree of freedom in χ^2 table
T	threshold used in deciding whether parameter is considered identifiable
β_0	margin used to see whether optimization proceeds based on CI evaluation
β_1	margin used to see whether optimization proceeds based on negative log likelihood

Table 1. Variables and Metaparameters

SI 2. Extended Results

SI 2.1. Comparison of Identifiability Metric

We considered different measures of Precision, based on Confidence Intervals, in devising our identifiability metric. The Precision measure (Eqn. 13) takes the ratio between confidence interval (Upper bound-Lower bound) and MLE; since the binding(k_{on}) and unbinding(k_{off}) rates span over several order of magnitudes, we take the log_{10} of standard uncertainty. By this definition, accuracy of the MLE is higher if MLE is numerically larger. However, as the MLE strongly depends on noise, if we sample data from a distribution, the MLE can take place anywhere between the upper and lower bound, meaning as long as with the same confidence interval, MLEs with larger numerical value is equally good/bad as MLEs with smaller numerical value. This contradicts with the definition of Precision. Furthermore, as the three parameters span over different ranges of parameter space, it is inappropriate to compare identifiability of the different parameters in this way.

To circumvent this issue, we proposed a different metric that is independent of MLEs, but only dependent on confidence interval (APM, Eqn. 14). The threshold depends on the nature of the parameter, for example, for gene switching rates, they span over several order of magnitude, and their effects are more nonlinear, the threshold of choice is 2, meaning if the width of confidence interval spans 100, the switching rate is considered unidentifiable. For k_{syn}, it spans over smaller order of magnitude, and its effect is more linear, the threshold of choice is 0.48, meaning the width of confidence interval spans over 3, the transcription rate is considered unidentifiable. Though one needs to decide what threshold to be used for each parameter based on the understanding of the model, nevertheless, it acts as a kind of normalization, and all parameters can be compared on the same ground. Though the two measures differ somewhat, there is broad qualitative similarity in their behavior over the parameter space. The resulting identifiability metrics of the parameter space is shown in Figure S1.

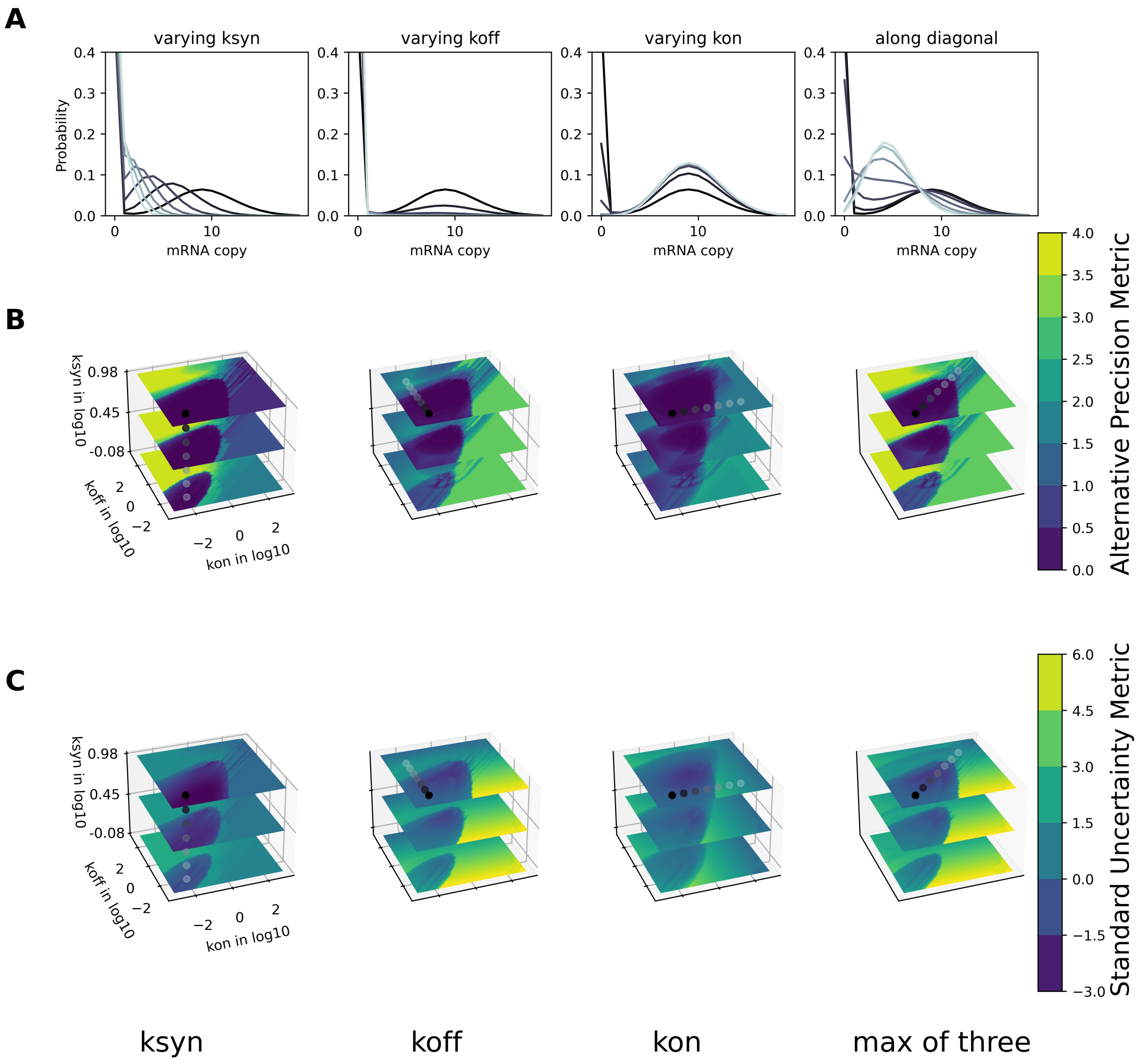

Fig. S1. Comparison of the two identifiability metrics: A) mRNA distributions of parameter sets, top row with full capture rate; B,C) Identifiability of each parameter, and the max of three parameters as the overall identifiability of the parameter set for two metrics, Alternative Precision Metric and Standard Uncertainty Metric respectively.

SI 2.2. Sensitivity analysis and comparison to distribution summary statistics

Here, the sensitivity vector of parameter i was computed as [1]:

$$\frac{d}{d\theta_i}\begin{bmatrix} P_{ss} \\ S_i \end{bmatrix} = \begin{bmatrix} A & 0 \\ C_i & A \end{bmatrix}\begin{bmatrix} P_{ss} \\ S_i \end{bmatrix} \tag{15}$$

where P_{ss} is the steady solution to the CME, and S_i is the sensitivity vector of parameter i. C_i is the derivative of A with respect to $log(\theta_i)$. $log(\theta_i)$ is chosen over θ_i because the identifiability metric is based on the width of the CI which is in log10 scale. For more detail, please refer to SI 3.3.

The computed S_i is summed along axes other than those dimensions that are experimentally observed; here, the sum is over the off/on promoter states, since only mRNA copy numbers, but not underlying promoter states, are experimentally measured. Singular decomposition of the sensitivity matrix S reveals how sensitive the steady state distribution of the stochastic dynamical

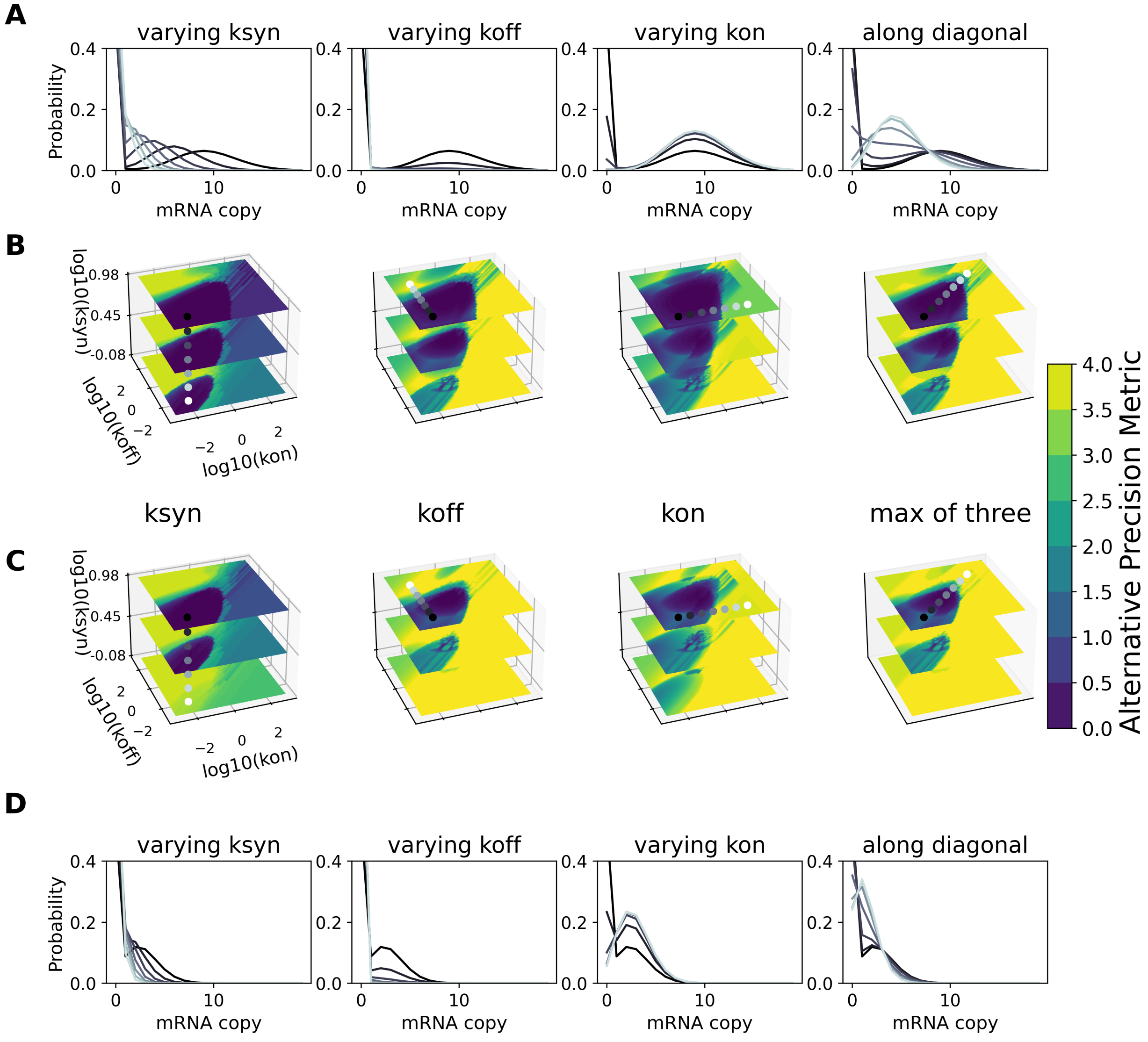

Fig. S2. Global *a priori* identifiability landscape over the entire studied parameter space at different capture rates for 10K cells. Same as Figure 3, except with a more stringent identifiability cutoff for calculation of the APM. Here, T values for $\{k_{\rm syn}, k_{\rm on}, k_{\rm off}\} = \{3, 10, 10\}$. (A,B) Results for 100% capture rate. (A) mRNA distributions for representative parameter sets. (B) Identifiability (measured by APM) at each ground-truth point in the 3D parameter space of each parameter separately (left three columns) and the overall identifiability (last column, maximum APM from all parameters). Distributions in (A) correspond to dots (grayscale color) in the corresponding 3D surfaces in (B). (C,D) Same as top rows, but with 0.3 experimental capture rate.

system is to the change of parameters. The smaller the minimum singular value, the less responsive the mRNA distribution to change of parameter values (with a zero value–singularity–indicating true structural non-identifiability).

We further explored how various RNA-distribution summary statistics varied as a function of the parameter values, reasoning that it could be useful if simple summary statistics (i.e., distribution shape measures) correlated with identifiability, thus providing an alternative means of *a priori* analysis. (Indeed, bimodality was seen to be a key feature, as described above). Two summary statistics that describe, at least in part, the distribution shape changes are the Fano factor σ^2/μ (often used as a measure of dispersion in gene expression) and the ratio of the centralized third-order moment (denoted $M3$) over the mean,

$$\sigma^2 = Var(X) = \sum_x (x-\mu)^2 * P_x \tag{16}$$

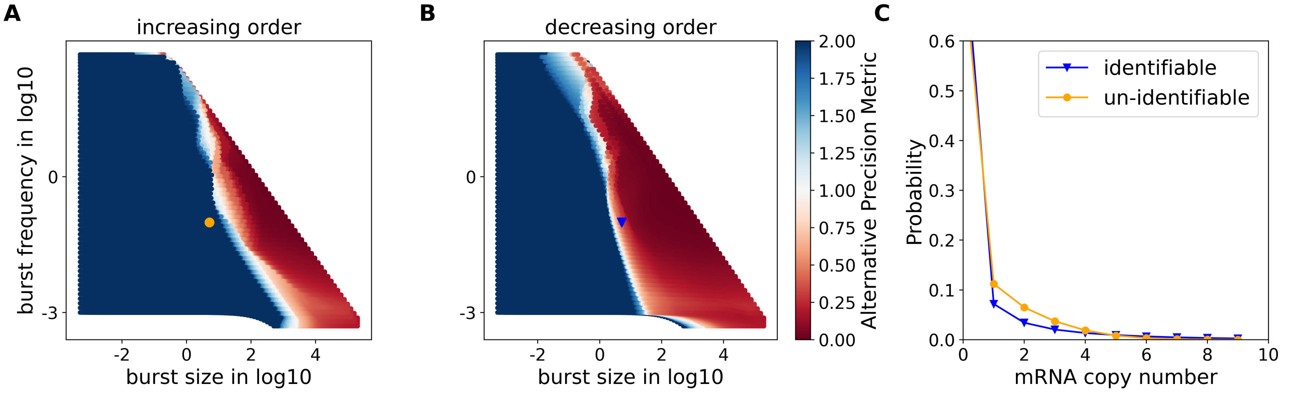

Fig. S3. Identifiability of transcriptional burst-size and burst-frequency via the telegraph model. The two representative parameter sets have the same burst dynamics (burst size: 5.13, burst frequency: 0.0975).(A,B) Scatter plots of APM (maximum over all three model parameters) from self-inference for 1000 cells plotted versus burst-frequency and burst-size. Information from 3D $\{k_{\text{on}}, k_{\text{off}}, k_{\text{syn}}\}$ (data as in Fig. 3) is projected onto the 2D space {burst-size, burst-frequency}. (There is a nonlinear relationship between the latter and the former, see Methods). Different projection methods are shown: (A) Increasing order, means that for a given point in the 2D plane, which maps to multiple points (computed APM values) in the 3D space, the highest APM value is visualized. (B) Decreasing order, means that the lowest APM value is visualized. Highlighted points correspond to two representative parameter sets, (k_{syn} : 20, k_{off} : 3.9, k_{on} : 0.1, orange point in (A)), (k_{syn} : 2.67, k_{off} : 0.52, k_{on} : 0.12, blue point in (B)). These two sets have the same burst size and burst frequency, but APM values of 1.86 (un-identifiable) and 0.448 (identifiable), respectively. (C) mRNA distributions of the two parameter sets, showing similar but different shapes.

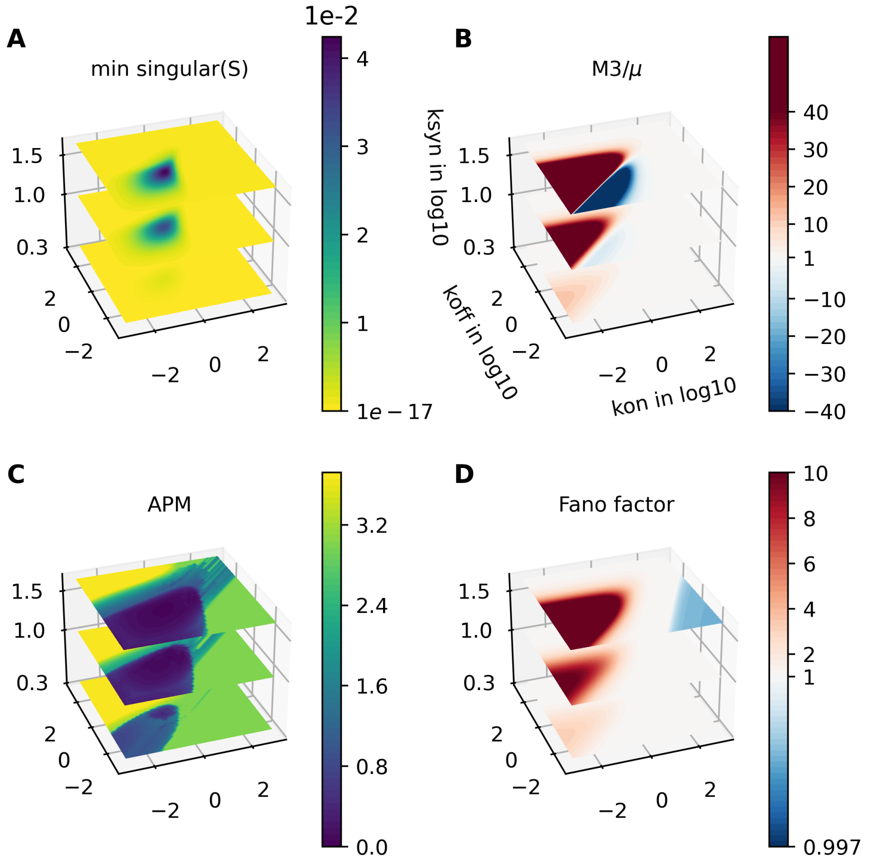

Fig. S4. Comparison of different approaches to *a priori* identifiability analysis over the parameter space. (A) Minimum singular value of the sensitivity matrix S, derived directly from the CME model; (B) Summary statistic $M3/\mu$, the centered 3rd moment divided by the mean; (C) Profile-Likelihood-based identifiability for comparison (maximum of the APM for 1000 cells and 100% capture, same as Fig. 3B, right); (D) Summary statistic Fano factor. Note that for B and D, a different color scale centered on 1 is used, to better highlight regions of non-Poisson distribution shape.

$$M3(X) = \sum_x (x-\mu)^3 * P_x \tag{17}$$

with μ the expectation value of the mRNA copy number. Division by μ ensures that Poisson-type distributions take the value of 1 for both metrics. $\frac{M3}{\mu}$ works like a higher order analog of the Fano factor, correlating with skewness of the distribution rather than dispersion. Positive values indicate skew towards low RNA copy numbers.

Both $M3/\mu$ (Fig. S4B) and Fano (Fig. S4C) generally have larger magnitudes in the inverted cone region of the parameter space where APM shows better parameter identifiability. Both measures take values close to 1 when either $k_{\text{off}} \gg k_{\text{on}}$ (gene mostly off, high cell number with 0 mRNA) or when $k_{\text{on}} \gg k_{\text{off}}$ (gene mostly on, Poisson distribution). This indicates that, in general, distributions with complex shapes (higher dispersion and/or skewness) can be predicted to have more identifiable bursting kinetics. However, both measures have limitations. For example, the Fano factor takes values close to 1 in the region of slow k_{off} and medium k_{on}, although there is moderately good identifiability in this region. This is because the distribution is bimodal, though with low probability in the 0-mRNA peak, which contributes little to the variance. $M3/\mu$ performs somewhat better in this same region, but is challenged when distributions are close to symmetric ($k_{\text{on}} \approx k_{\text{off}}$), albeit bimodal. When $k_{\text{off}} \gg k_{\text{on}}$ (distribution nearly unimodal at 0 mRNA, but with a small second peak affecting dispersion and skew while μ is low), both metrics can take high values despite rapidly decreasing identifiability in this region as one exits the inverted cone region. Additional summary statistics were also studied (Fig. S5).

SI 2.3. Neural Net-based prediction of parameter identifiability

Different distribution statistics measures were used as neural network input features. The list of statistics measures includes: Fano factor, mean (μ), variance (centralized second moment, M2), centralized third moment (M3), $\frac{M3}{\mu}$, $\frac{M3}{M2}$. Some of the statistic measures are visually more similar to the identifiability of parameter space computed from the computation pipeline in some region, but not entirely matching. Fig. S5 shows the different measures.

No single shape metric studied correlated perfectly with identifiability as quantified through the PL-based pipeline We reasoned that an identifiability predictor could be trained on our self-inference results, together with information on summary statistic features. Since identifiability is related to distribution shape, we asked whether it would be possible to rapidly assess identifiability for a given experiment-derived mRNA distribution (together with information on cell number and capture rate) without applying the full PL-based inference pipeline. In practice, only distributions predicted to be identifiable would then proceed downstream in the pipeline. This would reduce the amount of optimization required. However, with just summary statistics like moments and Fano factor, it is impossible to fully separate parameter sets with low and high APM at different capture rates and sample size with a simple linear regression model. To leverage the powerful generalization capability of deep learning, a neural network was trained to predict whether a distribution is identifiable or not. Summary statistics (those of Fig. S4 and Fig. S5) and sample size were used as input, and the APMs were used as output. The simulated data (60^3 distributions, at five sample sizes:$10^2, 10^3, 10^4, 10^5$) were used for training and testing at 7:3 ratio, respectively (training curve shown in Fig. S6A, data shown in Fig. S6 is from the deep learning model with the simplest multi-layered fully connected neural-net). When parameter sets were classified (identifiable, non-identifiable, very non-identifiable), 96.7% of identifiable parameter sets (in ground truth) were also predicted to be identifiable (Fig. S6B). Deeper analysis into the remaining sets that were wrongly predicted to be un-identifiable revealed that, in these cases, summary statistics used to train the neural net were not sensitive to small details in distribution shape that nevertheless promote identifiability and are somehow utilized in the PL-based pipeline, e.g., bimodality characterized by extremely low probability in the second (high copy number) peak, and bimodality that is degraded by down-sampling (i.e., to model low capture rate), but still gives the distribution a slightly modified shape. (See also discussion related to Fig. S4). Predictive capability of the neural net for the identifiability of each individual kinetic parameter is shown in Fig. S6C-E.

The neural network predicted 1945 genes in the SS3 data to be identifiable (that is, roughly twice as many genes than were truly identifiable, according to the PL pipeline). However, there was good overlap (877/970) between the set of identifiable genes (via PL) and those via the neural network. Similarly, for HUES64WT data sets, the neural network predicted 1384 genes to be identifiable, and 967 genes were considered identifiable according to both methods. Deeper analysis of the genes that were falsely predicted to have identifiable kinetics, according to the neural network, revealed that it is due in part to distribution features found in the real-world datasets that are under-represented in the simulated distribution library (namely, low mean and variance, whereby most probability lies at 0 mRNA with low probability of low copy number expression). The majority of these distributions have predicted APM between 1 and 2.5. These distributions mostly locate near the boundary of identifiable and un-identifiable region in Fig. S4.

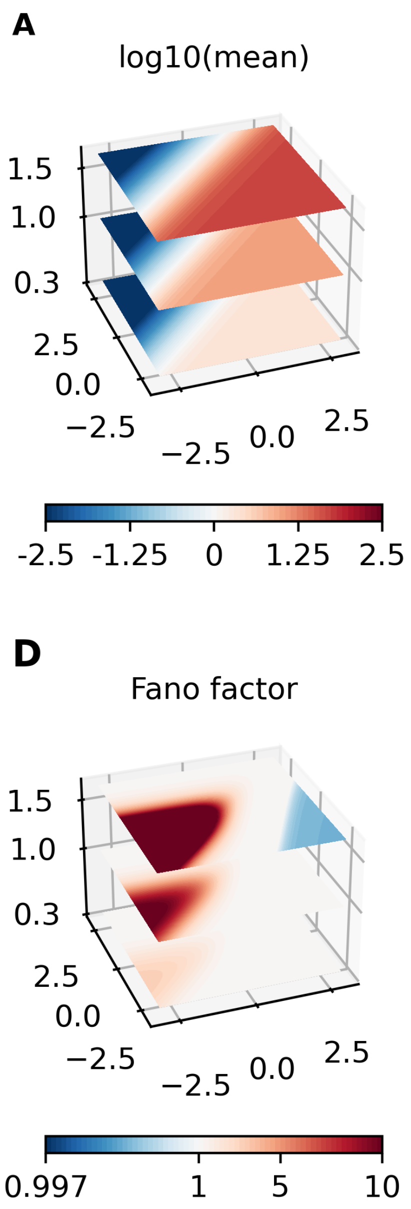

Fig. S5. Comparison of different statistical metrics of the parameter space: A) Mean (μ) in log10, B) Variance(M2) in log10, C) absolute value of centralized third order moment (M3) in log10, D) the Fano factor, E) $\frac{M3}{\mu}$, F) $\frac{M3}{M2}$.

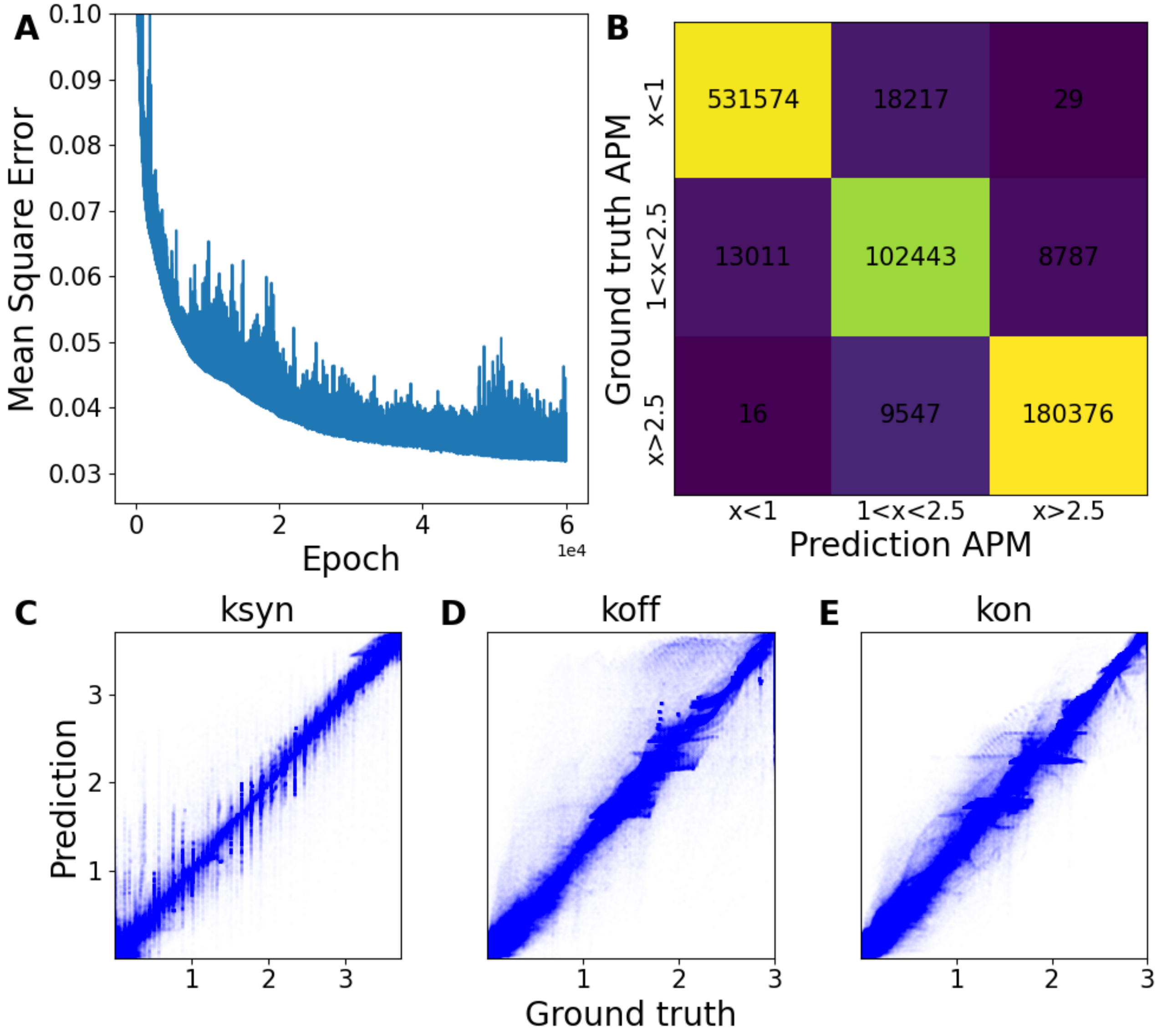

Fig. S6. Neural network prediction of parameter identifiability (as quantified by the APM) based on sets of statistical features or RNA distributions at 30% capture rate. A) Training curve of the neural network with testing mean square error; B) Confusion matrix for prediction of APM against ground truth. The number represents number of parameter sets. Larger values on the diagonal indicate better prediction. Parameter sets were classified into identifiable ($x < 1$, where x is the APM value), practically un-identifiable ($1 < x < 2.5$) and very practically un-identifiable ($x > 2.5$); C-E) Scatter plots of predicted APM value for each individual parameter over the full simulated library, versus the ground truth APM value. A good prediction should have the majority of dots near the diagonal.

SI 3. Supplementary Methods

SI 3.1. Transition Matrix Dimension Reduction

At steady state, left hand side of Eqn.2 is 0. Instead of solving the SVD of the whole transition matrix, for Eqn.2, it is rather convenient to solve it as linear system in block matrix form.

$$A_0 P_{G^*} + k_{\text{on}} I P_G = 0 => A_1 A_0 P_{G^*} + k_{\text{on}} A_1 P_G = 0$$

$$k_{\text{off}} I P_{G^*} + A_1 P_G = 0 => k_{\text{on}} k_{\text{off}} I P_{G^*} + k_{\text{on}} A_1 P_G = 0$$

$$(A_1 A_0 - k_{\text{on}} k_{\text{off}} I) P_{G^*} = 0$$

applying SVD on $A_1 A_0 - k_{\text{on}} k_{\text{off}} I$, P_{G^*} is obtained. However, it needs to be normalized and scaled. Unlike directly applying SVD on Eqn.2, the steady state distribution only needs to be normalized to 1. P_{G^*} needs to be further scaled by the probability of gene state G^*, which can be obtained from below:

$$k_{\text{off}} G^* = k_{\text{on}} G => \frac{G}{G^*} = \frac{k_{\text{off}}}{k_{\text{on}}} => \frac{G}{G^*} + 1 = \frac{k_{\text{off}}}{k_{\text{on}}} + 1 => G^* = \frac{k_{\text{on}}}{k_{\text{on}} + k_{\text{off}}}$$

Once P_{G^*} is obtained, P_G can be easily calculated. By doing in block fashion, solution is obtained by SVD on a matrix of size n, instead of SVD on a matrix of size 2n. This would largely increase computation efficiency, as the time complexity of SVD is $O(n^3)$. Fig. S7a illustrates how time increase in SVD changes against the matrix dimension. The increase in time grows exponentially as system dimension increases. The time taken for SVD operation are similar for operation in batch/tensor and in loop. However, significant difference in construction of the transition matrices between the two approaches. Fig. S8a demonstrate the accuracy of the solution compared to the one for solving full size transition matrix A. Since for a simple two-state switching system, there is no inverse calculation, there is practically no difference. Fig. S8b demonstrate the computation speedup by solving in blocks.

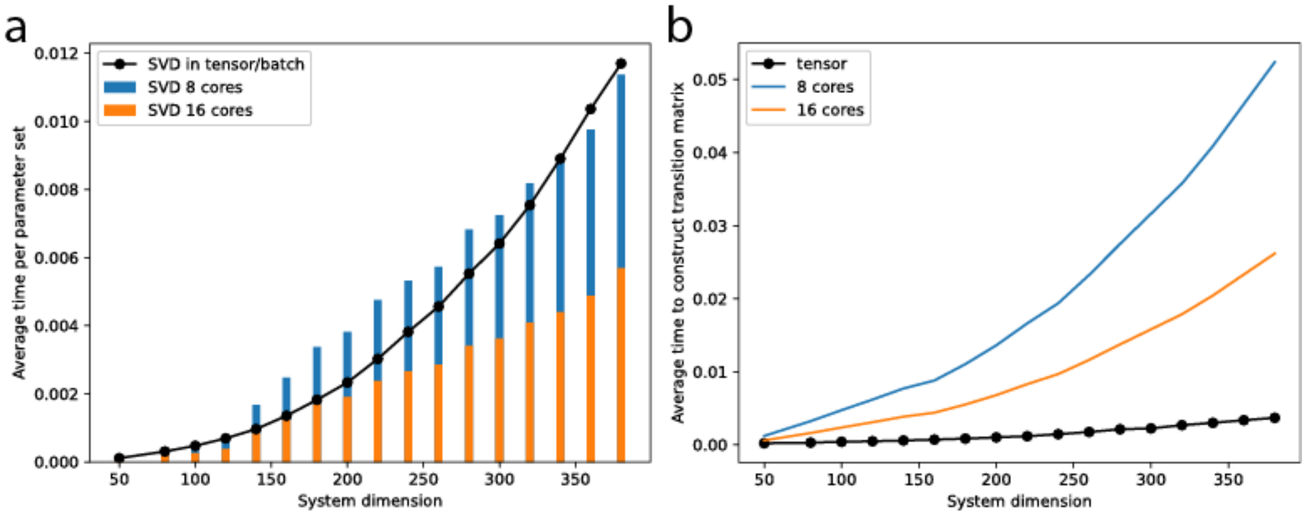

Fig. S7. (a) Computation efficiency of SVD at different system size and (b) Efficiency in constructing transition matrix in tensor/batch by a 8-cores,16 threads configuration; The blue and orange bars are operation in loop wise by a 8-cores,8 threads and 16-cores,16 threads setting running in parallel; The black dotted line is operation in batch-wise/tensor format in 16 threads setting.

In theory, regardless the number of gene states, the system can be reduced to SVD of matrix size n, with some linear algebra. For example, considering two promoter regions where two transcription factors can bind to, leading to four different gene states. Again, one can put different assumptions in the model. For example, (1) if both the promoter regions are bound, if there exist inhibitor, inhibitor wins, otherwise, the k_{ab} takes the larger value of k_a and k_b; (2) k_{ab} is the sum of k_a or k_b, etc.

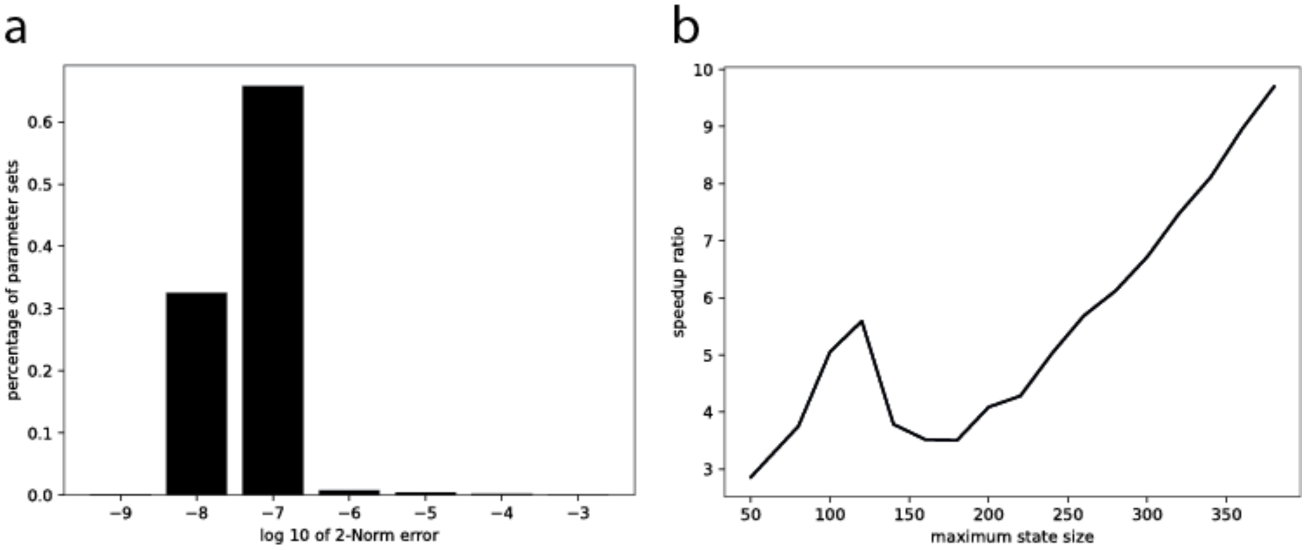

Fig. S8. Error and speedup comparison for two-state model;(a) Histogram of 2-norm error over the parameter space between solution of full-transition versus the one of block transition; (b) Speedup times of solving SVD on the same system with block approach relative to full-transition approach

$$\mathrm{G}_{00} \underset{\mathrm{f}_a}{\overset{\mathrm{h}_a}{\rightleftharpoons}} \mathrm{G}_{a0} \qquad \mathrm{G}_{a0} \underset{\mathrm{f}_b}{\overset{\mathrm{h}_b}{\rightleftharpoons}} \mathrm{G}_{\mathrm{ab}}$$

$$\mathrm{G}_{00} \underset{\mathrm{f}_b}{\overset{\mathrm{h}_b}{\rightleftharpoons}} \mathrm{G}_{0b} \qquad \mathrm{G}_{0b} \underset{\mathrm{f}_a}{\overset{\mathrm{h}_a}{\rightleftharpoons}} \mathrm{G}_{\mathrm{ab}}$$

$$(\mathrm{G}_{00} \xrightarrow{\mathrm{k}_0} \mathrm{G}_{00} + \mathrm{mRNA})$$

$$\mathrm{G}_{a0} \xrightarrow{\mathrm{k}_a \cdot \mathrm{k}_0} \mathrm{G}_{a0} + \mathrm{mRNA}$$

$$\mathrm{G}_{0b} \xrightarrow{\mathrm{k}_b \cdot \mathrm{k}_0} \mathrm{G}_{0b} + \mathrm{mRNA}$$

$$\mathrm{G}_{\mathrm{ab}} \xrightarrow{\mathrm{k}_{\mathrm{ab}} \cdot \mathrm{k}_0} \mathrm{G}_{\mathrm{ab}} + \mathrm{mRNA}$$

$$\mathrm{mRNA} \xrightarrow{\mathrm{k}_d} \varnothing$$

Again, the system can be represented in block matrix fashion:

$$\frac{d}{dt}\begin{bmatrix} P_{G_{ab}} \\ P_{G_{a0}} \\ P_{G_{0b}} \\ P_{G_{00}} \\ error \end{bmatrix} = \begin{bmatrix} A_0 & h_b I & h_a I & 0 & 0 \\ f_b I & A_1 & 0 & h_a I & 0 \\ f_a I & 0 & A_2 & h_b I & 0 \\ 0 & f_a I & f_b I & A_3 & 0 \\ B_0 & B_1 & B_2 & B_3 & 1 \end{bmatrix} \begin{bmatrix} P_{G_{ab}} \\ P_{G_{a0}} \\ P_{G_{0b}} \\ P_{G_{00}} \\ error \end{bmatrix} \tag{18}$$

Similarly, one can solve (18) block-wise with some linear algebra:

$$A_0 P_{G_{ab}} + h_b I P_{G_{a0}} + h_a I P_{G_{0b}} = 0 => P_{G_{a0}} = -h_b^{-1} A_0 P_{G_{ab}} - h_b^{-1} h_a P_{G_{0b}} \tag{19}$$

$$f_b I P_{G_{ab}} + A_1 P_{G_{a0}} + h_a I P_{G_{00}} = 0 => P_{G_{ab}} = -f_b^{-1} A_1 P_{G_{a0}} - f_b^{-1} h_a P_{G_{00}} \tag{20}$$

$$f_a P_{G_{ab}} + A_2 P_{G_{0b}} + h_b I P_{G_{00}} = 0 => P_{G_{ab}} = -f_a^{-1} A_2 P_{G_{0b}} - f_a^{-1} h_b P_{G_{00}} \tag{21}$$

$$f_a I P_{G_{a0}} + f_b I P_{G_{0b}} + A_3 P_{G_{00}} = 0 => P_{G_{a0}} = -f_a^{-1} f_b P_{G_{0b}} - f_a^{-1} A_3 P_{G_{00}} \tag{22}$$

(19) = (22):

$$f_a A_0 P_{G_{ab}} + f_a h_a P_{G_{0b}} = h_b f_b P_{G_{0b}} + h_b A_3 P_{G_{00}} => f_a A_0 P_{G_{ab}} = h_b A_3 P_{G_{00}} + (h_b f_b - h_a f_a) P_{G_{0b}} \tag{23}$$

Plug (21) to (23):

$$\begin{aligned} -A_0 A_2 P_{G_{0b}} - h_b A_0 P_{G_{00}} &= h_b A_3 P_{G_{00}} + (h_b f_b - h_a f_a) P_{G_{0b}} \\ => P_{G_{0b}} &= h_b (h_a f_a - h_b f_b - A_0 A_2)^{-1} (A_0 + A_3) P_{G_{00}} \end{aligned} \tag{24}$$

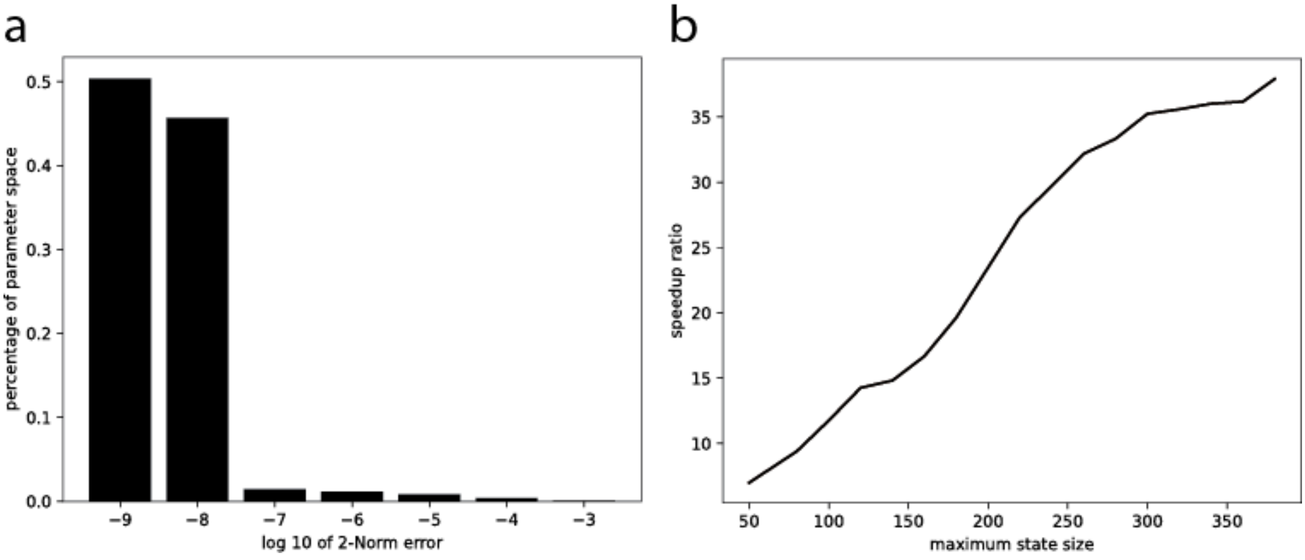

Fig. S9. Error and speedup comparison for four-state model;(a) Histogram of 2-norm error over the parameter space between solution of full-transition versus the one of block transition; (b) Speedup times of solving SVD on the same system with block approach relative to full-transition approach

(20) = (21):

$$f_a A_1 P_{G_{a0}} + f_a h_a P_{G_{00}} = f_b A_2 P_{G_{0b}} + f_b h_b P_{G_{00}} => f_a A_1 P_{G_{a0}} = f_b A_2 P_{G_{0b}} + (h_b f_b - h_a f_a) P_{G_{00}} \tag{25}$$

Plug (22) to (25):

$$\begin{aligned} -f_b A_1 P_{G_{0b}} - A_1 A_3 P_{G_{00}} &= f_b A_2 P_{G_{0b}} + (h_b f_b - h_a f_a) P_{G_{00}} \\ => P_{G_{0b}} &= f_b^{-1} (A_1 + A_2)^{-1} (h_a f_a - h_b f_b - A_1 A_3) P_{G_{00}} \end{aligned} \tag{26}$$

(24) = (26):

$$\left[f_b (A_1 + A_2) \underbrace{h_b (h_a f_a - h_b f_b - A_0 A_2)^{-1} (A_0 + A_3)}_{\text{dummy1}} - \underbrace{(h_a f_a - h_b f_b - A_1 A_3)}_{\text{dummy2}} \right] P_{G_{00}} = 0$$

Again, $P_{G_{00}}$ is obtained by performing SVD, and scaled to have sum with probability of state G_{00}:

$$G_{0?} * h_a = G_{a?} * f_a => G_{0?} = \frac{f_a}{f_a + h_a}$$

$$G_{?0} * h_b = G_{?b} * f_b => G_{?0} = \frac{f_b}{f_b + h_b}$$

$$G_{00} = G_{0?} G_{?0} = \frac{f_a f_b}{(f_a + h_a)(f_b + h_b)}$$

The rest of the probability distribution at other states can be calculated accordingly. Overall, for one-gene four-state model, one inverse matrix of size n is calculated and one SVD on matrix of size n is performed. As long as the transition matrix is written in the form of block based, and system states are categorized by gene states, the result of CME can be computed in a reduced form. It is much more efficient memory-wise and significantly faster compared to performing SVD on Eqn.18, matrix with size n*number of gene state. Fig.S9a shows the accuracy of the solving four-state system in block-wise compared to full transition matrix A. Fig.S9b demonstrates the computation acceleration.

To make use of efficiency of vectorized operation in modern computing, we put the transition matrix A of different parameter sets into a tensor format, and make all the algebra calculation batch wise. This would lead to significant speedup compared to loop-wise, so that the parameter space can be explored comprehensively and efficiently. The reduction in time is twofold: (1) Well-built package for tensor operation utilizes computation resources more efficiently(hyper-threading, multi-cores); (2) Vector operation is faster then looping. Fig.S7a shows the computation acceleration by performing SVD in tensor(batch) format. For system dimension up to about 340, performing in tensor/batch is no slower than in loop if not faster.

Though one can construct transition matrix by evaluate the minimum number of states from every set of kinetic parameters, this would also lead to significant overhead time. In contrast, by first categorizing the parameter sets into different group of maximum dimension systems, then constructing transition matrix batch-wise can lead to significant lower overhead computation, as shown in Fig.S7b.

SI 3.2. Mimicking Low Capture Rate in Sequencing

For inference of experimental data, since it is impossible to have 100% of the mRNA captured and amplified in scRNAseq, the actual mRNA distribution is downsampled with a certain capture rate, leading to an observed distribution with excessive zeros. To mimic such a process, the generated mRNA distribution library is modified with a binomial downsampling matrix with different capture rates[5]. Hence our modeling incorporates two types of technical error: limited sample size N and reduced capture rate. One can describe such binomial downsampling process as $Bi(i, x)$, the probability of observing i mRNA when there are x mRNAs in cell in a binomial distribution, and the observed/downsampled mRNA distribution can be found as:

$$\begin{bmatrix} P_0^{down} \\ P_1^{down} \\ P_2^{down} \\ \vdots \\ P_n^{down} \end{bmatrix} = \begin{bmatrix} Bi(0,0) & 0 & 0 & \dots & 0 \\ Bi(0,1) & Bi(1,1) & 0 & \dots & 0 \\ Bi(0,2) & Bi(1,2) & Bi(2,2) & \dots & 0 \\ \vdots & \vdots & \vdots & \vdots & 0 \\ Bi(0,n) & Bi(1,n) & Bi(2,n) & \dots & Bi(n,n) \end{bmatrix} \begin{bmatrix} P_0 \\ P_1 \\ P_2 \\ \vdots \\ P_n \end{bmatrix} = \begin{bmatrix} \sum_{i=0}^{0} Bi(i,0) * P_i \\ \sum_{i=0}^{1} Bi(i,1) * P_i \\ \sum_{i=0}^{2} Bi(i,2) * P_i \\ \vdots \\ \sum_{i=0}^{n} Bi(i,n) * P_i \end{bmatrix} \tag{27}$$

Here, we assumed that mRNAs from different genes share the same probability to be captured/amplified during sequencing process.

SI 3.3. Sensitivity Computation

In the context of CME, we can compute sensitivities with a little modification (constraint of sum of S_{k_i}=0):

$$\frac{d}{dt} \begin{bmatrix} P(\overrightarrow{X}|k_i) \\ S(\overrightarrow{X}|k_i) \end{bmatrix} = \begin{bmatrix} A & 0 \\ C_i & A \end{bmatrix} \begin{bmatrix} P \\ S_{k_i} \end{bmatrix}$$

where S_{k_i} is the sensitivity of model output with respect to parameter k_i, and C_i is the partial derivative of A with respect to parameter k_i. Sensitivity matrix only represents the local effect, however, the effect of each parameter on the resulting mRNA distribution in non-linear. Further, identifiability of parameter set is quantified by the width of confidence interval in log10 scale, therefore, instead of directly calculating sensitivity with respect to parameter value, a better approach is to calculate with respect to the log of parameter:

$$\frac{\delta A}{\delta ln(k_i)} = \frac{\delta A}{\delta k_i} * \frac{\delta k_i}{\delta ln(k_i)} = \frac{\delta A}{\delta k_i} * k_i$$

With a known P, S_{k_i} is obtained relatively easily with condition that sum of S_{k_i} is 0 (total change/flow of probability is 0). One can construct the sensitivity matrix from the sensitivity vector respective to each parameter, and by performing SVD, the smallest singular value and its corresponding unitary vector represent the magnitude and direction where each parameter reflects to the minimal change to the corresponding parameter/least sensitive. In stead of using the dimensionless sensitivity where the sensitivity vector is normalized by $\frac{\theta_k}{P}$, we kept the absolute value here. A comparison of the minimum singular value of sensitivity with respect to parameter value and the log of parameter value is shown in Figure S10.

References

1. Fox, Z. R. and Munsky, B. (2019). The finite state projection based fisher information matrix approach to estimate information and optimize single-cell experiments. *PLoS computational biology*, 15(1):e1006365.
2. Gillespie, D. T. (1977). Exact stochastic simulation of coupled chemical reactions. *The journal of physical chemistry*, 81(25):2340–2361.
3. Munsky, B. and Khammash, M. (2006). The finite state projection algorithm for the solution of the chemical master equation. *The Journal of chemical physics*, 124(4).
4. Murphy, S. A. and Van der Vaart, A. W. (2000). On profile likelihood. *Journal of the American Statistical Association*, 95(450):449–465.
5. Tang, W., Jørgensen, A. C. S., Marguerat, S., Thomas, P., and Shahrezaei, V. (2023). Modelling capture efficiency of single cell rna-sequencing data improves inference of transcriptome-wide burst kinetics. *Bioinformatics*, 7(39).
6. Wilks, S. S. (1938). The large-sample distribution of the likelihood ratio for testing composite hypotheses. *The annals of mathematical statistics*, 9(1):60–62.

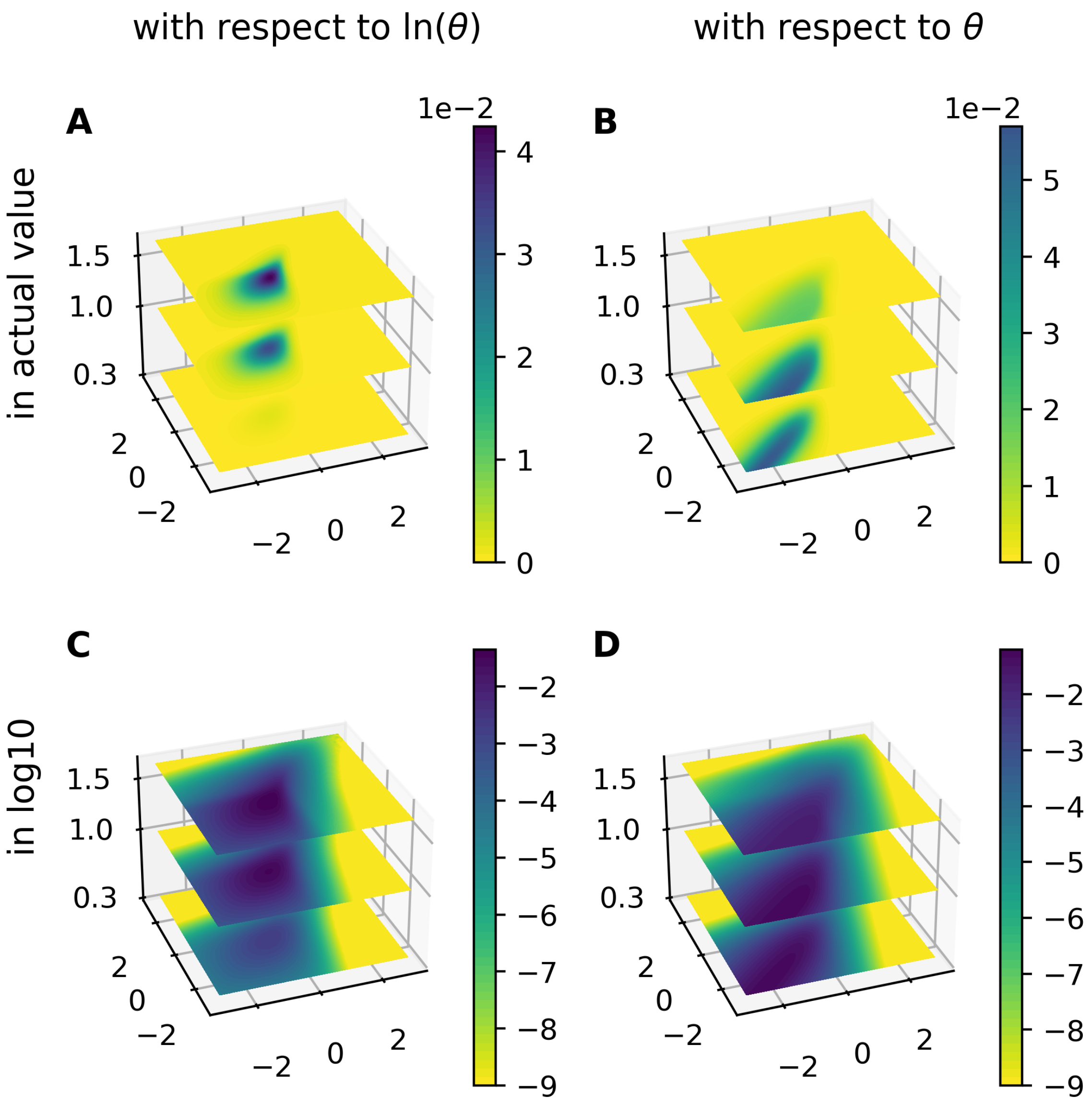

Fig. S10. Comparison of minimum singular value of sensitivity with respect to log of parameter (first row) and with respect to parameter (second row), the log10 of singular value are shown in the second column, to better shown the order change of singular value
